# Combining Classical and Molecular Approaches Elaborates on the Complexity of Mechanisms Underpinning Anterior Regeneration

**DOI:** 10.1371/journal.pone.0027927

**Published:** 2011-11-18

**Authors:** Deborah J. Evans, Suthira Owlarn, Belen Tejada Romero, Chen Chen, A. Aziz Aboobaker

**Affiliations:** Centre for Genetics and Genomics, Queen's Medical Centre, University of Nottingham, Nottingham, United Kingdom; University of Sheffield, United Kingdom

## Abstract

The current model of planarian anterior regeneration evokes the establishment of low levels of Wnt signalling at anterior wounds, promoting anterior polarity and subsequent elaboration of anterior fate through the action of the TALE class homeodomain PREP. The classical observation that decapitations positioned anteriorly will regenerate heads more rapidly than posteriorly positioned decapitations was among the first to lead to the proposal of gradients along an anteroposterior (AP) axis in a developmental context. An explicit understanding of this phenomenon is not included in the current model of anterior regeneration. This raises the question what the underlying molecular and cellular basis of this temporal gradient is, whether it can be explained by current models and whether understanding the gradient will shed light on regenerative events. Differences in anterior regeneration rate are established very early after amputation and this gradient is dependent on the activity of Hedgehog (Hh) signalling. Animals induced to produce two tails by either *Smed-APC-1(RNAi)* or *Smed-ptc(RNAi)* lose anterior fate but form previously described ectopic anterior brain structures. Later these animals form peri-pharyngeal brain structures, which in *Smed-ptc(RNAi)* grow out of the body establishing a new A/P axis. Combining double amputation and hydroxyurea treatment with RNAi experiments indicates that early ectopic brain structures are formed by uncommitted stem cells that have progressed through S-phase of the cell cycle at the time of amputation. Our results elaborate on the current simplistic model of both AP axis and brain regeneration. We find evidence of a gradient of hedgehog signalling that promotes posterior fate and temporarily inhibits anterior regeneration. Our data supports a model for anterior brain regeneration with distinct early and later phases of regeneration. Together these insights start to delineate the interplay between discrete existing, new, and then later homeostatic signals in AP axis regeneration.

## Introduction

The process of regeneration is widely observed across metazoan phyla; with most clades having members that possess some ability to replace lost or damaged tissues as adults. In recent years the study of regenerative mechanisms has become increasingly tractable in a number of experimental systems that all hold promise for informing how we might one day regenerate lost or damaged human tissues [1]. Among these the planarians represent a simple system within which to establish a detailed description of how the processes of regeneration are controlled [2], [3]. Planarian regeneration is the result of proliferation and differentiation of pluripotent planarian adult stem cells (pASCs), classically called neoblasts [4]. These cells can replace all missing planarian tissues after almost any amputation using signals present in remaining tissue [2], [3]. After injury, pASCs are induced to proliferate above normal basal rates in two well-characterised mitotic maxima at 6 and 48 hours of regeneration (hR) [5], [6]. pASC progeny gather at the wound site to form an unpigmented regeneration blastema and these cells differentiate to replace missing distal structures as more progeny migrate from the post-blastema region into the blastema [2], [7]. Remaining tissues remodel to restore scale and proportion [2], [8]. We are interested in the mechanisms that control anteroposterior (A/P) axis formation and in particular the regeneration and restoration of pattern and function of the brain and anterior tissues after amputation.

We now know that correct A/P axis specification is dependent on correct Wnt and Hedgehog (Hh) signalling to establish regenerative polarity [9], [10], [11], [12], [13], [14], [15], [16], [17]. For example, in the model species *S. mediterranea*, loss of Wnt activity leads to ectopic regeneration of anterior structures at all wounds and a gradual homeostatic anteriorisation of the whole axis if animals are left unwounded [12], [13], [14]. Conversely, ectopic activation of Wnt signalling leads to suppression of anterior regeneration, and the regeneration of ectopic tails at anterior facing wounds after decapitation [12], [13]. During A/P regeneration correct maintenance of wound induced Wnt signalling at posterior blastemas requires active Hh signalling [15], [16], [17], and ectopic Hh signals can also lead to the regeneration of posterior structures at anterior facing wounds [16], [17]. These RNAi analyses of Wnt and Hh signalling components along with detailed consideration of the expression patterns of Wnt and Hh pathway genes suggest that Wnt signalling acts in a gradient over the A/P axis to specify the correct fates during regeneration and homeostasis [3], [10], [11], [18]. However, we currently know little about the downstream events that control the elaboration of regenerative polarity and A/P patterning, or exactly how existing polarity is read, to ensure that axes are correctly re-specified. We do know that high Wnt signals lead to the post-transcriptional inhibition of Smed-Prep and the activity of this transcription factor is required to promote brain regeneration in the anterior blastema [19].

Many planarian species can regenerate a head from a decapitation made anywhere along the A/P axis, however the rate of regeneration decreases more posteriorly [20]. Morgan was the first to concern himself with the phenomenon of how A/P polarity is re-established in a transverse section [21]. When the head and tail are cut from an animal, a head will regenerate from the anterior facing wound, whilst a tail will regenerate from the posterior facing wound [8]. Morgan's narrow transverse sections occasionally produced two-headed animals he named “Janus heads” [22]. This suggests that a minimal distance between the anterior and posterior blastemas is required for correct A/P specification. Child concluded that these Janus heads were the result of a lack of polarity differences within the existing tissue and that polarity consists of a dynamic gradient along the axis [23]. Although Morgan agreed with this gradient hypothesis [24], he proposed that the gradient was due to a structural or substance difference over the axis whereas Child [25] proposed that polarity was due to a metabolic gradient [26], [27]. These were the first gradient hypotheses proposed to explain the phenomenon of A/P axis specification, of which, Morgan's hypothesis fits well with what we know about Wnt signalling in planarian regeneration. We anticipated that an attempt to describe this classical gradient observation with molecular details would advance our understanding of the orchestration of anterior regeneration.

Here, we show that the widely used laboratory model planarian *S. mediterranea* has a clear gradient of anterior regenerative rate along the A/P axis [6]. Our experiments reveal that hedgehog signalling is controlling this temporal difference. Our experiments to investigate the gradients effect on anterior fate and brain regeneration confirm that early brain structures always differentiate at anterior wound sites even in animals that will ultimately regenerate two tails [28]. We show that these brain structures form from cells that have progressed through S-phase of the cycle before amputation and commit to brain fate regardless of Wnt or Hh signalling levels within the first 16 hours after decapitation. Later in regeneration we find that animals that regenerate two tails through ectopic Hh signalling are able to homeostatically regenerate a new anterior, and brain, from central tissues around the pharynx. Together our data elaborate on the existing models of A/P axis specification, the events underpinning brain regeneration and predict the existence of an alternate A/P gradient distinct from Wnt signalling.

## Results

### Pre-pharyngeal fragments of *S. mediterranea* regenerate heads more rapidly than post-pharyngeal fragments

**Figure 1:**
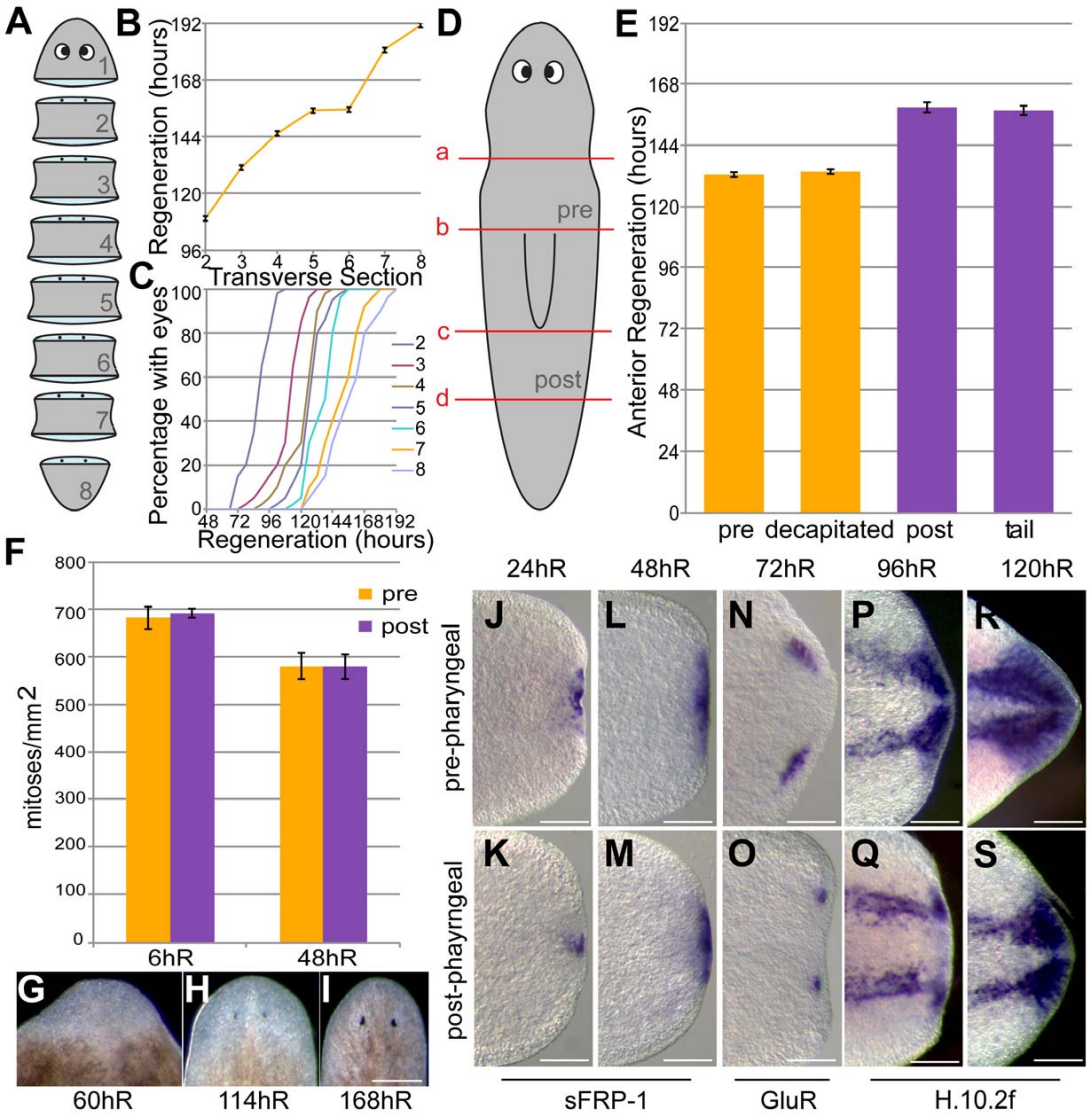
The Anterior Regeneration rate along the S. mediterranea A/P axis. (A) Schematic showing eight transverse sections (1–8) of equal length. (B) Plot of the average time (hours) taken for regenerating fragments from each transverse section (2–8) to regenerate two visible photoreceptors, showing the graded anterior regeneration rate over the A/P axis of *S. mediterranea* (n = 40). (C) The percentage of fragments along the A/P axis with two eyes at six hour time intervals, showing that the kinetics with which photoreceptors appear is broadly the same between transverse sections. (D) A schematic illustration of transverse incisions to investigate the effects of the extent of remaining posterior tissue by anterior wound sites. Double incisions at a and b or c and d create pre-pharyngeal (pre) and post-pharyngeal (post) regenerates respectively, with starting sizes of 1.4 mm. Single incisions at a or c create decapitated or tail regenerates respectively. (**E**) The average time in hours taken for, pre-pharyngeal (pre), decapitated, post-pharyngeal (post) and tail pieces to regenerate two visible photoreceptors indicates that it is the level of anterior amputation that determines differences in the rate of anterior regeneration, not the amount of remaining tissue (n = 40) (F) Proliferation assay of pre-pharyngeal (pre) and post-pharyngeal (post) transverse sections at 6hR and 48hR (mean number of mitotic cells, error bars are +/- SEM, n = 12) shows that different rates of anterior regeneration are not due to spatial differences in cell proliferation. (G–J) *Smed-sFRP-1* (n = 18), (K,L) *Smed-GluR* (n = 18) and (M to P) *H.10.2f* (n = 20) expression is shown in anterior blastemas of pre-pharyngeal and post-pharyngeal regenerates at 24 to 120 hR suggesting that temporal differences in regeneration between pre-pharyngeal and post-pharyngeal fragments are established very early in regeneration in *S. mediterranea*. Scale bars 250 µm.

We first wished to establish the temporal dynamics of anterior regeneration in our laboratory strain of the model planarian *S. mediterranea*. We created eight positionally matched transverse sections (Figure 1A) along the A/P axis of individual animals (n = 40, all animals 1.5 cm +/−500 µm in length) and observed the rate of anterior regeneration. Animals were observed in 6 hr time windows and the time at which two photoreceptors were clearly visible recorded (Fig 1B). We analysed the average time at which each level of transverse section regenerated two visible photoreceptors (Figure 1B) and the percentage with two eyes at each 6 hr time window (Figure 1C). We observed a decline in the rate of photoreceptor regeneration in more posterior sections, as has been previously described for many, but not all, planarian species capable of brain regeneration [20]. Further decapitation experiments that either left all posterior tissues intact or removed them showed this effect was independent of the amount of tissue remaining behind the anterior amputation or the presence of a posterior blastema (Figure 1D and E). To exclude effects of subjective judgments each sample was scored blindly on both live images and then again independently with captured images (Figure 1 G, H and I are representative images of negative, positive and final images respectively). Together our data confirm that pre-pharyngeal sections are able to regenerate anterior photoreceptors more rapidly than more post-pharyngeal sections in *S. mediterranea*, and that the position of the anterior amputation along the A/P axis dictates differences in anterior regeneration rates.

### Temporal differences in anterior regeneration are independent of proliferation and are established early in regeneration

To obtain an initial description of the cellular and molecular differences that account for the increased rate of regeneration in more anterior pieces we investigated the timing of expression of anterior and brain markers and the rate of cell division. We ruled out differential rates of proliferation as a possible cause of different anterior regenerative rates using anti-phosphohistone H3 Serine 10 (anti-H3P-Ser10) staining at the two mitotic maxima [5] to label proliferating cells in pre-pharyngeal and post-pharyngeal transverse sections (Figure 1F, n = 12). We next looked at the expression of the anterior fate marker *Smed-sFRP-1* (Figure 1J-M, n = 18 at 24hR and 48hR), the brain specific marker *Smed-GluR* (Figure 1N and O, n = 18) and the CNS marker *H.10.2f* (Figure 1P-S, n = 20). We observed that pre-pharyngeal pieces regenerate their brains and integrate with the existing CNS more rapidly than post- pharyngeal pieces, as shown by both *Smed-GluR* and *H.10.2f* expression (Figure 1N-S). This difference in the extent of anterior regeneration is also observed by greater expression of *Smed-sFRP-1* in the anterior blastema at 24hR and can be used as a proxy for the early temporal progression of anterior regeneration. These data are in agreement with the observed rate of photoreceptor regeneration and classical data implicating the formation of the brain as a prerequisite for the induction of photoreceptor regeneration [6], [27]. Therefore we conclude the temporal difference in anterior regeneration between pre-pharyngeal and post- pharyngeal fragments is not caused by differential proliferative rates and is established early in regeneration after decapitation.

### *Smed-hedgehog* is required for temporal differences in anterior regeneration along the A/P axis

Previous work has implicated both the Wnt and Hh signalling pathways in controlling regeneration of the A/P axis by promoting posterior polarity and fate [9], [11], [12], [13], [14], [15], [16], [17], [18]. These pathways represent candidates for providing the information that controls differences in the rate of anterior regeneration. Given that we see that a temporal difference in regeneration is established very early we hypothesised that pre-existing gradients of these signals along the A/P axis may be responsible. We reasoned that if pre-existing gradients of Wnt and/or Hh signalling were the underlying cause of differences in regenerative rates then modulation of their activity should affect anterior regeneration rates.

We performed both *Smed-hedgehog(RNAi)* and *Smed-βcatenin-1(RNAi)* (see Figure S1 for RNAi regime), and observed regeneration of both pre- and post-pharyngeal pieces. In all cases *Smed-βcatenin-1(RNAi)* animals regenerated posterior facing heads [12], [13], [14]. However, we observed a clear and significant difference in the incidence of the previously reported tailless *Smed-hedgehog(RNAi)* phenotype in pre- and post-pharyngeal fragments [16], [17]. All pre-pharyngeal fragments displayed a tailless phenotype whereas only 70% of post-pharyngeal fragments were tailless, suggesting a greater requirement for *Smed-hedgehog* in more anterior fragments (n = 40/40, n = 14/14, n = 10/10 tailless in pre-pharyngeal fragments in 3 separate experiments and n = 28/40, n = 8/13, n = 6/10 in post-pharyngeal fragments in 3 separate experiments).

**Figure 2:**
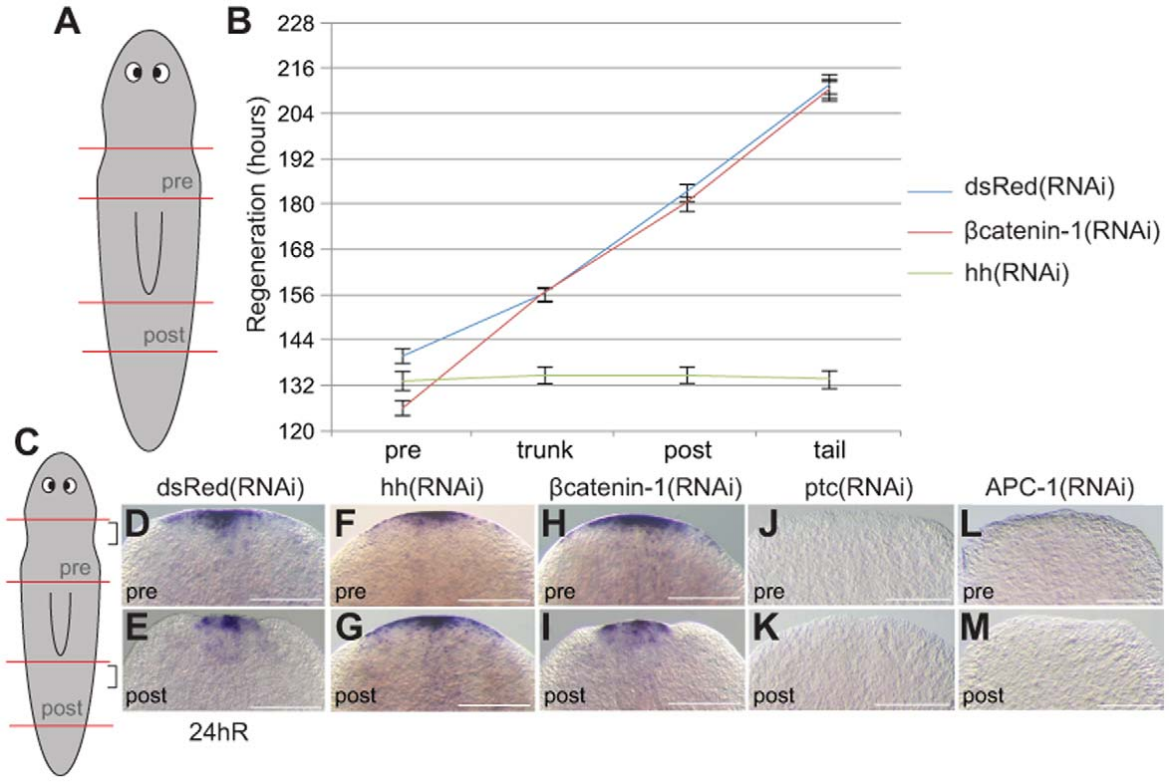
*Smed-hedgehog* is required for differences in A/P regenerative rates. (A) Animals were amputated to produce pre- and post-pharyngeal pieces and trunk and tail pieces. (B) Photoreceptor regeneration rates were compared in control, *Smed-βcatenin-1(RNAi)* and *Smed-hedgehog(RNAi)* worms for pre- and post-pharyngeal pieces and trunk and tail pieces. (C) *Smed-sFRP-1* expression was compared between pre- and post-pharyngeal pieces in (D,E) control, (F,G)*Smed-hedgehog(RNAi)* worms, (H,I)*Smed-βcatenin-1(RNAi)*, (J,K) *Smed-ptc(RNAi)* and (L,M) *Smed-APC-1(RNAi)*. Scale bars 250 µm.

We also observed the rate of photoreceptor regeneration as a proxy of anterior regeneration rate (see Figure S1 for RNAi experiment, Figure 2A and B). *Smed-βcatenin-1(RNAi)* had no effect on the anterior regeneration gradient (Figure 2B). However, *Smed-hedgehog(RNAi)* completely eliminated any difference between more anterior and more posterior cut sites. Post-pharyngeal anterior regeneration rates increased to match those of pre-pharyngeal pieces (Figure 2B). These data suggest that in fact pre-existing differences in levels of hedgehog signalling or differences established very shortly after amputation are responsible for differences in regenerative rate.

To test this further we used the *Smed-sFRP-1* marker to investigate the early establishment of anterior fate in pre- and post-pharyngeal fragments (Figure 2D and E). We interpret punctate and/or antero-medial expression (Figure 2E) as representative of temporally earlier stages of anterior regeneration than expression that has expanded laterally (Figure 2D). *Smed-βcatenin-1(RNAi)* animals maintained a temporal difference in the extent of *Smed-sFRP-1* expression, with pre-pharyngeal fragments showing more extensive *Smed-sFRP-1* staining around the anterior blastema than post-pharyngeal fragments at 24 hR (Figure 2H and I, 10/10 animals) in a pattern similar to injected controls (Figure 2D and E, 8/8 animals). This correlates with the observation that no difference was observed in eye regeneration rates.

*Smed-hedgehog(RNAi)* resulted in loss of the difference in *Smed-sFRP-1* expression, with equivalent early expression observed between pre- and post-pharyngeal fragments (Figure 2F and G, 28/30 animals across 3 independent experiments). This correlates with the observation that anterior and posterior fragments of *Smed-hedgehog(RNAi)* animals regenerate their photoreceptors at the same rate.

We also performed *Smed-ptc(RNAi)* and *Smed-APC-1(RNAi)* as controls as knockdown of these genes leads to ectopic Wnt and Hh signalling, ectopic posterior fate and loss of *Smed-sFRP-1* expression (Figure 2 J-M and Figure S2 for internal in situ controls on head pieces).

Taken together these data suggest that the anterior regenerative rate is controlled by a pre-existing hedgehog signalling gradient. This difference is not due to differences in stem cell proliferation (Figure S3). While promoting posterior fate hedgehog signalling may also act to initially inhibit anterior regeneration at more posteriorly positioned anterior facing wound sites. The fact that *Smed-hedgehog(RNAi)* also results in a higher incidence of the tailless phenotype in more anterior fragments also supports the existence of lower pre-existing levels of hedgehog signalling in anterior regions. Alternatively it remains possible that *Smed-hedgehog(RNAi)* may have pleiotropic functions effecting early *Smed-sFRP-1* expression and the later formation of photoreceptors and neither effect is reflective of anterior regeneration events in between.

### Temporal differences in early brain regeneration are not dependent on *Smed-hedgehog*

**Figure 3:**
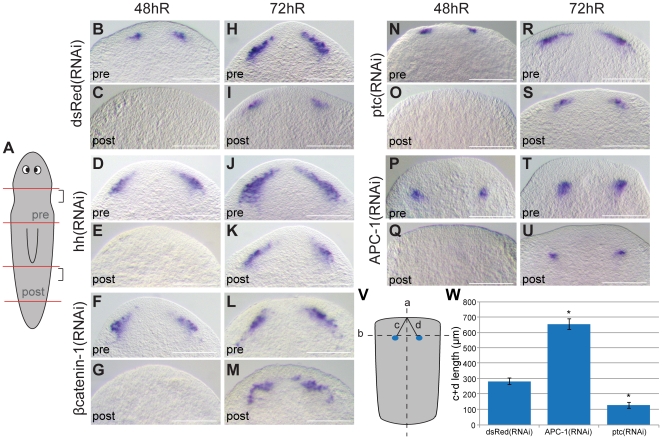
Early brain structures form independently of regenerative polarity at anterior amputation sites. (A) Brain regeneration was monitored with either *Smed-GluR* or *Smed-Gpas* (shown) expression in pre- and post-pharygngeal pieces. (B–G and N–Q) at 48 hR and (H–M and R–U) 72 hR in control *dsRed(RNAi)* (B,C,H and I), *Smed-hedgehog(RNAi)* (D, E, J and K), *Smed-βcatenin-1(RNAi)* (F,G L and M), *Smed-ptc(RNAi)* (N,O,R and S) and *Smed-APC-1(RNAi)* (P,Q,T and U) animals. Scale bars 250 µm. (V) A schematic illustrating measurements of the position of brain structures. Line ’a’ is down the lateral midline. The line ‘b’ was generated by joining the most anterior points of stained brain structures. Line ‘c’ and ‘d’ were created from the anterior midline to the anterior of each brain structure**.** (W) Average length of c plus d for *dsRed(RNAi), Smed-APC-1(RNAi)* and *Smed-ptc(RNAi)* regenerates demonstrate that *Smed-APC-1(RNAi)* animals regenerate brains at a greater distance from the anterior margin than *dsRed(RNAi)* or *Smed-ptc(RNAi)* regenerates. In all measurements 72hR pre-pharyngeal regenerates were used (n = 20 per RNAi experiment, error bars are +/− 1 SEM of the mean c+d length, P<0.05 for *dsRed(RNAi)* vs. *APC-1(RNAi)*, P<0.01 *ptc(RNAi)* vs. *APC-1(RNAi)*), t-test, two tailed.

Given that hedgehog signalling is required for differences in anterior regenerative rate as reflected by both expression of an early anterior marker and by tracing the regeneration of photoreceptors we also predicted this effect would be reflected in brain regeneration. We used the markers *Smed-GluR* and *Smed-Gpas* [29] to follow brain regeneration in pre and post-pharyngeal pieces (Figure 3A). We observed that brains regenerated more rapidly as measured by the extent of *Smed-GluR* or *Smed-Gpas* at 48 and 72 hours of regeneration in both *Smed-hedgehog(RNAi)* and *Smed-βcatenin-1(RNAi)* compared to control animals (Figure 3B–M). More importantly differences in anterior regenerative rate of brain fated tissues are in fact maintained in both *Smed-hedgehog(RNAi)* and *Smed-βcatenin-1(RNAi)* at 48 hours (Figure 3B–M). We interpret this finding as indicating that the temporal control of early brain regeneration may not be subject to the same Hh dependent control as early anterior polarity as reflected by *Smed-sFRP-1* expression or later Wnt and Hh dependent elaboration of the brain and subsequent photo-receptor regeneration. We also observed that both *Smed-APC-1(RNAi)* and *Smed-ptc(RNAi)* animals always regenerated *Smed-GluR* and *Smed-Gpas* positive early brains at anterior wounds (Figure 3N–U).

### Early brain regeneration is independent of regenerative polarity and reveals a difference between *Smed-APC-1(RNAi)* and *Smed-ptc(RNAi)* phenotypes

We proceeded to investigate in greater detail the finding that *Smed-APC(RNAi)* and *Smed-ptc(RNAi)* animals still regenerate early brains at anterior wounds. An array of phenotypes for *Smed-APC-1(RNAi)*, ranging from hypermorphic cyclopic animals, reminiscent of weaker classes of *Smed-prep(RNAi)* phenotypes to animals with two tails, has previously been reported. We found that injection doses of 1 µg/µl of *Smed-APC-1* dsRNA led to the formation of animals with two tails, ectopic posterior structures (Figure S4, n = 32/32), loss of *Smed-sFRP-1* expression at anterior blastemas (Figure 2L and M, Figure S2 for internal control, n = 20/20) and expansion of the posterior marker *Smed-fz-4* (Figure S5, n = 12/12) as previously reported [12], [16]. We also observed that all animals developed early *Smed-GluR* and *Smed-Gpas* positive brain structures in the post-blastema (Figure 3P,Q,T and U, n = 32/32 at 48 h and n = 26/26 at 72 h) as previously reported [29]. These brain structures appeared, like control *dsRed(RNAi)* animals (Figure 3B, C, H, and I), earlier in more anterior pieces, suggesting that differences in anterior regenerative rate along the A/P axis are still present even with respect to these ectopic early brains (Figure 3P vs. Q and T vs. U).

We also performed *Smed-ptc(RNAi)* which also leads to the formation of two tails, ectopic posterior structures (Figure S4, n = 48/50), loss of *Smed-sFRP-1* expression at anterior blastemas (Figure 2J and K, Figure S2 for internal control, n = 24/24) and expansion in the expression of the posterior marker *Smed-fz-4* (Figure S5, n = 12/12) as previously described [16], [17]. In these animals we also observed ectopic *Smed-GluR* and Smed-Gpas positive brain structures (Figure 3N,O,R,S, n = 28/28 at 48hR and 27/28 at 72hR). Again, brain structures appeared earlier in more anterior pieces, suggesting that differences in regenerative rate along the A/P axis are still present even with respect to these early ectopic brains (Figure 3 N vs O and R vs S). Together these data show that anterior wounds regenerate early brain tissue even when they will go on to ultimately form posterior fated blastemas and new posterior tissues. In addition these early brains still display an anterior regenerative rate.

**Figure 4:**
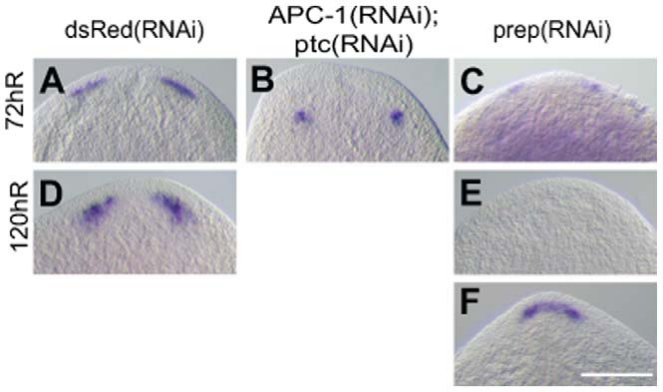
*Smed-prep(RNAi)* and *Smed-APC-1(RNAi);Smed-ptc(RNAi)* early brain phenotypes. Brain regeneration monitored in regenerating tail pieces in (A, D) control (B) *Smed-APC-1(RNAi);Smed-ptc(RNAi)* and (C, E and F) *Smed-prep(RNAi)*). Double *Smed-APC-1(RNAi);Smed-ptc(RNAi)* worms phenocopy single *Smed-APC-1(RNAi)* worms. *Smed-prep(RNAi)* worms have early brains, albeit reduced (C), later at 120 hR *Smed-prep(RNAi)* animals either have no detectable brain structure remaining (E) or have a single fused brain structure (F). Scale bar represents 250 µm.

In *Smed-ptc(RNAi)* animals, early brains appeared to be clearly positioned in the anterior blastema whereas in *Smed-APC-1(RNAi)* animals brain structures differentiated in the post-blastema (Figure 3R vs T and S vs U). As an independent test of early brain position we measured their position in control and RNAi animals in relation to the medial anterior tip of animals (Figure 3V and W, n = 20 for each condition). This confirmed that *Smed-ptc(RNAi)* animals have significantly more anteriorly positioned brain structures than *Smed-APC-1(RNAi)* animals, in a similar average position to controls. Performing double *Smed-APC-1(RNAi);Smed-ptc(RNAi)* phenocopied the *Smed-APC-1(RNAi)* phenotype by limiting ectopic brain structures to the post-blastema (see Figure 4B).

Our data are in agreement with a previous study that also observed polarity independent brain regeneration and reveal that brains form independently of regenerative polarity and ultimate regenerative fate [28]. However, elaboration of the brain and further anterior regeneration are inhibited by ectopic Wnt and Hh signals, and early brains fail to expand as posterior fate is promoted. To see if formation of these early brains was a property of the unusual regenerative scenario caused by inducing an ectopic anterior Wnt/Hh signal or if this also occurred independently of Wnt/Hh signal modulation we utilised the *Smed-prep(RNAi)* phenotype. Previous work has shown that correct anterior and brain regeneration requires Smed-Prep activity in regions of low Wnt activity [19]. We wished to see if *Smed-prep* might be required for early polarity independent brain formation. While *Smed-prep(RNAi),* as previously described, led to the complete loss or severe reduction of brain structures at 12 days of regeneration (dR), early brain structures were detected at 72hR (Figure 4A vs C, n = 10/10), in a similar position to those observed for *Smed-ptc(RNAi)* animals. Later, by 120hR, these early brain structures either disappeared (Figure 4E, n = 12/20) or were replaced by miss-patterned ganglia in the process of fusing (Figure 4F remaining 8/20), as previously described for the *Smed-prep(RNAi)* phenotype later in regeneration. This suggests that early polarity independent brain regeneration is not a peculiarity caused by ectopic high levels Wnt or Hh signalling, but also occurs in the absence of the instructive *Smed-prep* signal. We also note that early *Smed-prep(RNAi)* brain structures were always smaller than those observed in either *Smed-ptc(RNAi)* or *Smed-APC-1(RNAi)* worms.

Together our data show that while Hh signalling does affect differences in the rate of anterior regeneration along the A/P axis an early phase of brain regeneration proceeds regardless of ultimate regenerative polarity controlled by Wnt and Hh or elaboration permitted by *Smed-prep* activity. Instead, we find that that early brain regeneration occurs at anterior facing wounds that will ultimately have posterior fate. This early ectopic brain regeneration also displays differential rates along the A/P axis.

### The late phenotypes of *Smed-APC-1(RNAi)* and *Smed-ptc(RNAi)* reveal homeostatic regulation of axial identity to restore a full A/P axis

**Figure 5:**
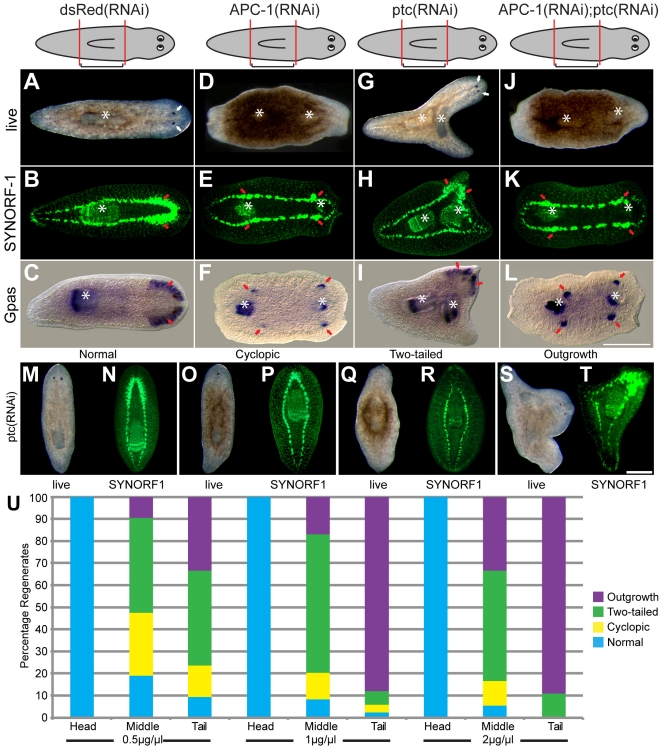
Brain structures differentiate homeostatically in peri-pharyngeal regions in animals that regenerate two tails. Bright field, *Smed-Gpas*, and anti-SYNORF1 stained images of (A–C) control *dsRed(RNAi),* (D–F) *Smed-APC-1(RNAi)*, (G–I) *Smed-ptc(RNAi)* and (J–L) double *Smed-APC-1(RNAi);Smed-ptc(RNAi)* trunk pieces at 40 days of regeneration. White arrows indicate photoreceptors in control *dsRed(RNAi)* heads and in *Smed-ptc(RNAi)* outgrowths, red arrows indicate cephalic ganglia in *dsRed(RNAi)* heads, either side of *Smed-APC-1(RNAi)* and *Smed-APC-1(RNAi);Smed-ptc(RNAi)* pharynges and in *Smed-ptc(RNAi)* outgrowths, whilst white stars identify the single pharynx in control *dsRed(RNAi)* and double pharynges in *Smed-APC-1(RNAi), Smed-ptc(RNAi)* and *Smed-APC-1(RNAi);Smed-ptc(RNAi)* regenerates. (M,N), Bright field and anti-SYNORF1 images of regenerating head fragments, (O,P) Example of “cyclopic” animal, (Q,R) example of “two-tailed” animal and (S,T) anterior outgrowth as phenotypes of *Smed-ptc(RNAi)* worms. (U) Distribution of different phenotypes caused by increasing doses of *Smed-ptc(RNAi)* doses. Scale bars represent 250 µm.

We wished to observe what happened to early ectopic brain structures later during regeneration in animals that regenerated two tails. Control *dsRed(RNAi)* animals regenerated normally with anterior bilobed cephalic ganglia together with two photoreceptors and a central pharynx (Figure 5A–C). However, we observed a two-tailed phenotype with small brain structures in all *Smed-APC-1(RNAi)* trunk regenerates (Figure 5D–F and Figure S6, n = 50/50). In all trunk *Smed-APC-1(RNAi)* animals we observed the regeneration of a second pharynx in opposite orientation to the original pharynx with paired brain structures around this new pharynx. We found that 76% of these animals displayed peri-pharyngeal brain structures around both the new and the old pharynx (Figure S6). These findings are in line with the previous report that also observed peri-pharyngeal brain structures after *Smed-APC(RNAi)* and *Smed-axins(RNAi)* [29].

*Smed-ptc(RNAi)* animals regenerated trunk and tail pieces with centrally positioned outgrowths containing well developed brain structures and eventually photoreceptors (Figure 5G–I, 35% of trunk pieces, 90% of tail pieces n = 50, at the highest dose of dsRNA injections). This process led to the establishment of animals with a new A/P axis, albeit with two tails (Figure S7) and involved the re-orientation of the newly regenerated pharynx, creating tails with one, and trunks with two, pharynges (see Figure S7 for phenotypic progression). We showed that this later *Smed-ptc(RNAi)* phenotype was dose dependent with increased concentrations of dsRNA injection leading to an increased frequency of new A/P axis formation (Figure 5M–U). It is possible previous studies did not detect this phenotype due to incomplete knockdown or because they did not observe later stages of regeneration.

Like the early *Smed-APC-1(RNAi);Smed-ptc(RNAi)* phenotype, the later phenotype of these animals phenocopy *Smed-APC-1(RNAi)* animals (Figure 5J-L), suggesting that Wnt signalling is downstream of Hh signalling during A/P axis specification events, in agreement with other studies [16], [17]. Given that here and in previous studies [16], [17] neither *Smed-ptc* or *Smed-hedgehog* have homeostatic phenotypes (i.e. generated without amputation) we suggest that the effect of Hedgehog signalling may be specific to regeneration and act through modulation of a Wnt signalling gradient [29].

Together these data suggest that planarians are able to homeostatically regulate A/P axis identity as long as Wnt signalling components are left intact, as is the case for animals that have two tails due to *ptc(RNAi)*. In addition our data provide evidence that ventral nerve cord (VNC) regions around the pharynges may have the most anterior positional identity in animals with two tails induced by ectopic Wnt signalling as pASCs differentiate into brain tissue in these regions.

### Adult planarian stem cells that have progressed through S-phase at the time of amputation form the early brain structures

We next wished to know the source of the cells forming early brain structures. Our data thus far implicate a Wnt and Hh independent mechanism in existing tissue in directing the differentiation of pASCs to form early brain structures at the anterior wound site, although the ultimate position of these structures is Wnt activity dependent (Figure 3W).

**Figure 6:**
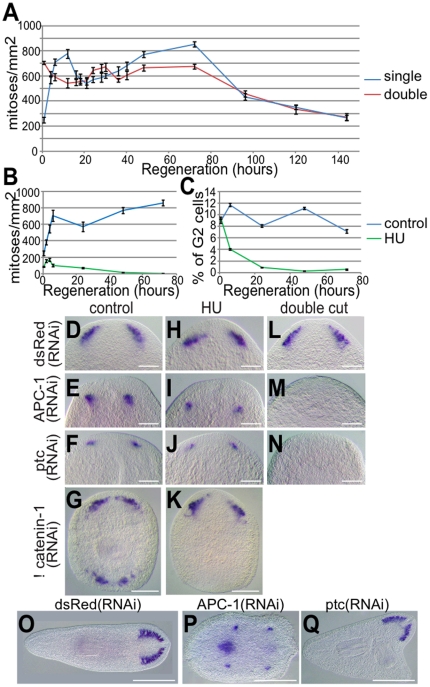
Polarity independent brain structures form from stem cells that have progressed through S-phase during the first 16 hours of regeneration. (A) Proliferation assay of single and double amputated trunk pieces at different times of regeneration indicate double cuts produce a decrease in the amplitude of both characteristic mitotic peaks and a delay in the first mitotic peak (mean number of mitotic cells, error bars are +/− 1 SEM of the mean, n = 12 at each time point). (B) Proliferation assay and(C) FACS analysis of the percentage of G2 cells in control (blue, n = 12) and HU (green, n = 10) treated regenerating pieces at different times of regeneration, confirms the efficiency of HU treatment to block pASCs progressing through S-phase of the cell cycle. *Smed-GluR* expression shows brain regeneration in (D,H,L) the anterior of control *dsRed(RNAi),* (E,I,M) *Smed-APC-1(RNAi),* (F,J,N) *Smed-ptc(RNAi)* and (G,K) anterior and posterior of *Smed-βcatenin-1(RNAi)* trunks pieces at 72 hR. *Smed-GluR* expression is observed in anterior blastemas of control *dsRed(RNAi), Smed-APC-1(RNAi), Smed-ptc(RNAi)* and anterior but not the posterior of *Smed-βcatenin-1(RNAi)* HU treated trunks at 72hR implicating pASCs that have progressed through S-phase at the anterior wound site as the source of cells for early brain regeneration. (L–N) *Smed-GluR* expression reveals brain regeneration in anterior blastemas of control *dsRed(RNAi)* but not *Smed-APC-1(RNAi)* and *Smed-ptc(RNAi)* double amputated trunks at 72hR after a second cut revealing a 16 hour window in which early Wnt/Hh independent brain regeneration occurs. (O,P,Q) *Smed-GluR* expression is shown in double amputated trunks at 40dR indicating that brain regeneration in control *dsRed(RNAi), Smed-APC-1(RNAi)* and *Smed-ptc(RNAi)* phenotypes can occur independently of the Wnt and Hh independent phase of the first 16 hours. Scale bars represent 100 µm in panels D, E, F, H,I ,J ,L, M and N and 250 µm in G and K and after 40 dR in O, P and Q.

Given that Wnt signals control ultimate A/P axis polarity, we reasoned that this early mechanism must eventually be affected by ectopic Wnt signals. To define the time at which ectopic Wnt and Hh signalling would be able to block the formation of ectopic brain structures after amputation, we performed simple double cutting experiments. We amputated animals once and then again just behind the regenerating blastema (see Figure S8 for schematic). This procedure resulted in a decrease in the amplitude of both characteristic mitotic peaks and a delay in the first mitotic peak (Figure 6A). We found that re-cutting 16 hours after initial amputation was sufficient time to completely block the formation of polarity independent brains, as detected by *Smed-GluR* or *Smed-Gpas* expression, in both *Smed-APC-1(RNAi)* and *Smed-ptc(RNAi)* animals (Figure 6L-N, 10/10 animals in for *Smed-APC-1(RNAi)* and *Smed-ptc(RNAi)*, 7/7 for controls). Thus, at this time the double cut anterior blastema has the properties of a posterior blastema, with respect to early brain regeneration. This suggests the signals in existing tissue directing early brain regeneration may have been reprogrammed by 16hR. Control, *dsRed(RNAi)* animals form normal brains after double cuts, showing this is not an effect of the procedure (Figure 6L, n = 7/7).

We continued to observe *Smed-APC-1(RNAi)* and *Smed-ptc(RNAi)* double cut animals and found that they developed later peri-pharyngeal brain structures and full anterior outgrowths with brains respectively, whilst control *dsRed(RNAi)* animals regenerated as normal (Figure 6O, n = 12/12 P, n = 7/10 and Q, n = 8/8). Thus we conclude the later phenotypes we observe of new anterior regeneration and peri-pharyngeal brain structures are actually not dependent on early polarity independent brain formation specified in the first 16hR. Instead they represent independent attempts to establish anterior identity in response to ectopic anterior Wnt and Hh signalling after this time.

The observation that double cuts stop the formation of early brains in *Smed-APC-1(RNAi)* and *Smed-ptc(RNAi)* backgrounds prompted us to consider the cellular source of early brain structures. As early ectopic brain structures form all along the A/P axis we hypothesised that brain structures were likely to be formed from uncommitted pASCs or progeny in response to decapitation. These cells would be capable of reading their position as being at the most anterior point in remaining tissue and be initially non-responsive to ectopic Wnt and Hh signals with respect to differentiation decisions (but not migration to he blastema in the case of *Smed-APC(RNAi)* animals). Given that 16 hours is after the first proliferative peak and this peak consists of many cells in S-phase, G2 and M at the time of wounding (Figure 6B, C and Figure S9) we hypothesised that brain structures may be formed from pASCs at later phases of the cell cycle at the time of amputation.

To investigate this hypothesis further we used hydroxyurea (HU) to block the transition of pASCs through the cell cycle at S-phase. We confirmed the efficacy of this treatment by monitoring mitotic activity (Figure 6B, C and Figure S9) and analysing the cell cycle by FACS analysis (Figure 6B, C and Figure S9), and looked at early brain formation. We found that HU treated animals stopped cells entering mitosis and displayed a rapid depletion of cells in G2 or M as expected (Figure 6B and C, Figure S9). Control, *Smed-APC-1(RNAi)* and *Smed-ptc(RNAi)* HU treated animals were all still able to regenerate brain structures (Figure 6H, n = 20/20 , I, n = 19/19 and J, n = 18/18, Figure S10 for in situ controls), however they all failed to elaborate these structures and subsequently died. These experiments suggest that brain structures are established from pASC cells and/or uncommitted post-mitotic progeny that have progressed through S-phase of the cell cycle. To attempt to further narrow down the cellular source of early brain structures we looked to see if we observed early brain regeneration after colchicine treatment, which stops cells transiting through mitosis. We found that animals did not make brain structures, further implicating pASCs that have progressed through S-phase at the time of amputation as the source of brain structures (Figure S11), although it is alternatively possible that colchicine treatment blocks differentiation of post-mitotic undifferentiated progeny.

Together these data suggest that early Wnt independent brain structures form from pASCs that have progressed through S-phase or uncommitted post-mitotic progeny positioned at anterior wounds. These cells are able to read their position in remaining tissue and those in the most anterior post-blastema region begin to differentiate into brain cells, independently of regenerative polarity. To further test these new insights we decided to look at ectopic anterior structures that form when Wnt signalling is abrogated by *Smed-βcatenin-1(RNAi)* animals that form anterior structures at all amputation sites. We reasoned that if signals instructing early brain structures are specifically directed at anterior stem cells progressed through S-phase, then ectopic anterior structures caused by *Smed-βcatenin-1(RNAi)* would not have a contribution from this cell population at posterior wounds present at the time of amputation. This predicts that *Smed-βcatenin-1(RNAi)* HU treated animals will form early anterior brain structures but no brain structures in the posterior. We observed that while *Smed-βcatenin-1(RNAi)* animals produced brains in both blastemas, HU treated *Smed-βcatenin-1(RNAi)* animals only produced brains in anterior blastemas (Figure 6G, n = 10/10, Figure 6K, n = 10/10). These data show that ectopic anterior structures in the posterior caused by abrogation of Wnt signalling form independently of the novel early brain regeneration process we have uncovered here.

## Discussion

### Time graded regeneration of early brain structures along the A/P axis occurs independently of polarity signals

*S. mediterranea* regeneration displays clear temporal differences in the rate of anterior regeneration along the AP axis (Figure 1). We observe that the dynamics of photoreceptor regeneration rates are broadly similar between amputations at different axial levels after an initial posterior latency in their appearance and differences in expression of the anterior marker *Smed-sFRP-1* as a proxy for the progress of anterior regeneration can be detected very early. We note that abrogation of Wnt signalling by *Smed-βcatenin-1(RNAi)* results in no difference in anterior regenerative rates or *Smed-sFRP-1* expression between anteriorly and posteriorly positioned amputations. However, abrogation of *Smed-hedgehog(RNAi)* signalling removes differences in regenerative rates and early differences in Smed-sFRP-1 expression. Together our data suggest that differences in anterior regeneration rate are established very early after amputation and are dependent on a gradient of hedgehog signalling. The finding that we also observe a difference in the *Smed-hedgehog(RNAi)* “tailless” phenotype between anterior and posterior pieces also supports the existence of a pre-existing gradient.

Our observation that early brain structures regenerate at all anterior amputations, including those that will regenerate to form tail structures, reveals an early phase of brain regeneration that is Wnt and Hh independent. This has also been recently described by a study investigating the role of Smed-Axin molecules [28]. We also observe that these brain structures still regenerate more rapidly in more anterior pieces. Together these data provide compelling evidence of a Wnt and Hh independent activity gradient contributing to an early phase of Wnt and Hh independent brain regeneration. Earlier studies may have failed to report this as they assessed the expression of anterior markers and not brain markers, or looked too late to observe these early anterior brain structures in *Smed-APC-1(RNAi)* and *Smed-ptc(RNAi)* animals. Although, we note that *Yawaza et al* [*17*] observe *Dj-ndk* expression in the early anterior blastemas of *Dj-ptc(RNAi)* worms and do suggest that *Dj-ptc(RNAi)* and ectopic Hh signalling leads to a transition from anterior to posterior fate.

### Brain structures form from cells through S-phase

**Figure 7:**
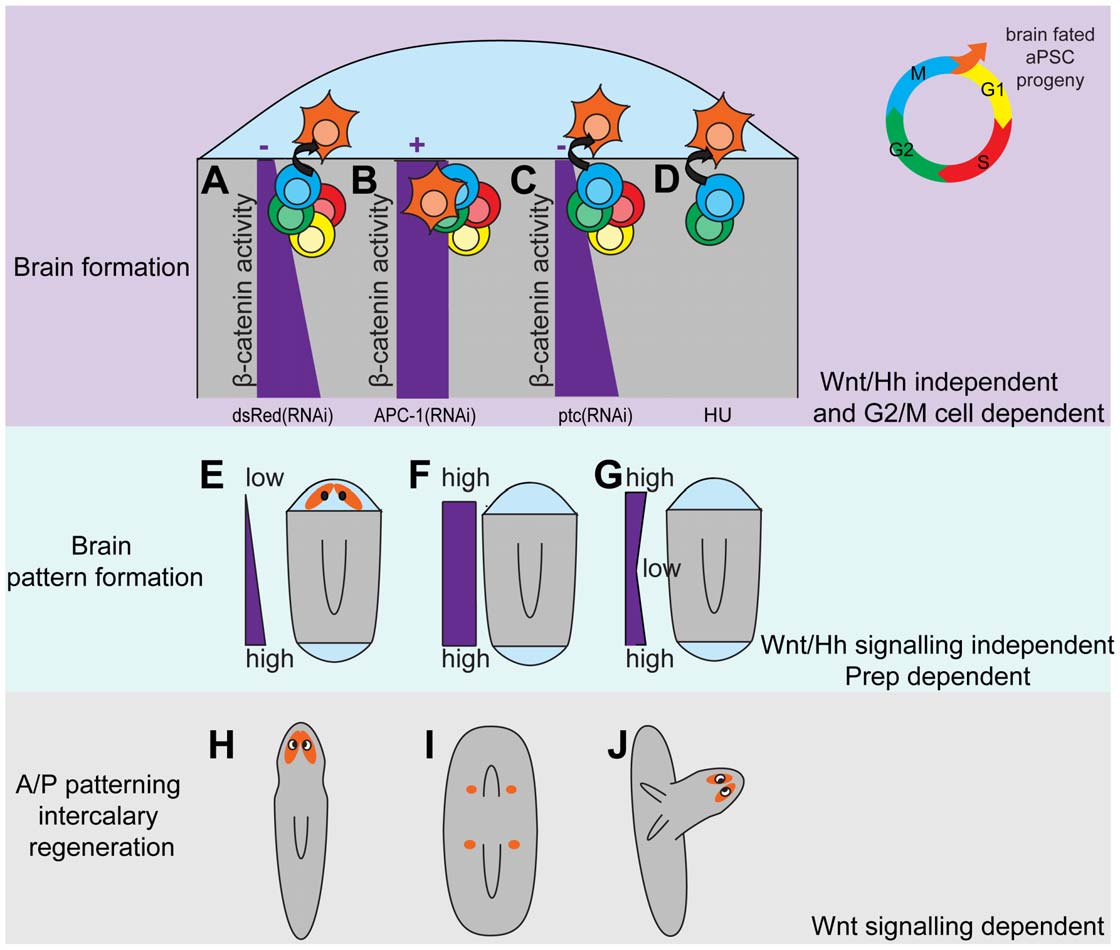
Summary of brain regeneration phases revealed by studying the classically described anterior regenerative rate of *S. mediterranea.* (A–D) Schematic illustration of the first 16 hours of brain regeneration as revealed by experiments in this study and later brain regeneration events (E–J) as a result of ectopic Wnt and hedgehog signaling. Early Wnt/Hh insensitive brain structure regeneration is revealed by the regeneration of brain structures in all RNAi conditions (A–C). The difference in the position of brain structures between (B) *Smed-APC-1(RNAi)* and (C) *Smed-ptc(RNAi)* animals reveals that ectopic Wnt signaling works before or very early after amputation to dictate regenerative polarity while ectopic Hh signaling works later through promoting later ectopic Wnt signaling. (D) HU treated animals form early brains in all RNAi conditions suggesting that cells that differentiate to form early brains have progressed through S-phase of the cell cycle. (E) Later elaboration of the brain structures in regions of low Wnt/Hh signals and active Smed-Prep in control animals. (F) Brain structures in central body regions appear in the peri-pharyngeal region in *Smed-APC-1(RNAi)* animals but (I) do not elaborate, presumably because high levels of Wnt signaling inhibit anterior fate. (G and J) Intercalary regeneration of a new head can occur in central body regions of *Smed-ptc(RNAi)* animals with two tails if Wnt signaling components are left intact and a gradient can be re-established. Orange, brain fated tissue; Light blue, blastema tissue; Yellow cells, G1 phase pASCs; Red cells, S phase pASCs; Green cells, G2 phase pASCs; Blue cells, M phase pASCs; Orange cells, brain fated pASC progeny; Purple bars, assumed gradient of β-catenin activity across the regenerating A/P axis.

While ectopic Wnt/Hh signalling cannot block the formation of anterior early brain structures initially after amputation, we find that after 16 hours no brain cells will regenerate after re-amputation. This suggests that ectopic Wnt/Hh signals are able to reprogram stem cells and/or their progeny by this time point. This 16 hour time point in regeneration lies between the two characteristic proliferative peaks as measured by anti-H3P-Ser10 staining [5]. The first of which will include a significant contribution from cells that were in G2/M at the time of amputation (see Figure S10). Using a combination of RNAi experiments, and HU and colchicine treatments we have shown that uncommitted stem cells through S-phase are a likely source of early brain polarity independent brain structures (Figure 7A–D). We cannot currently discount an effect of colchicine treatment on the differentiation of post-mitotic pASC progeny, so cannot entirely exclude these cells as a possible source. We also cannot exclude some contribution from stem cells earlier in the cell cycle but given the size of brain structures is consistent between *Smed-APC-1(RNAi);Smed-ptc(RNAi)* with and without HU treatment (Figure 6) we suggest that this contribution, if any, is minimal.

By combining HU treatment with *Smed-βcatenin-1(RNAi)* we show that ectopic brain ganglia and anterior fate at posterior blastemas do not involve this early phase of regeneration. These data also suggest that the early phase of brain regeneration we have uncovered maybe be redundant for the ultimate restoration of anterior structures, and that low Wnt signalling activity alone is sufficient to instruct correct anterior regeneration, agreeing with the proposal that anterior fate may be the default state of blastemas. Redundancy of regenerative mechanisms in planarians has not been described by direct experimental means before. One intriguing possibility is that the early brain regeneration phase uncovered here may represent part of the mechanism for beginning the regeneration of anterior structures during post-pharyngeal fission. Before the fission process this would be a region of relatively high Wnt signal and thus refractory to the de novo regeneration of anterior structures. This remains to be tested by looking at early brain regeneration during the process of fission with molecular markers in asexual animals.

### Differences between the effects of ectopic Wnt and Hh signalling are revealed by both early and later brain regeneration phenotypes

The observations that early brain structures differentiate in different relative positions in *Smed-APC-1(RNAi)* and *Smed-ptc(RNAi)* indicates a difference in the timing of fate specification in the blastema (Figure 7 A–D). In *Smed-APC-1(RNAi)* animals brain structures are limited to the post blastema (Figure 7B), while they are positioned within the blastema of *Smed-ptc(RNAi)* animals (Figure 7C). This suggests that while Wnt signals act homeostatically and after amputation in regeneration, Hh signals only act after amputation and during regeneration to instruct A/P polarity. In agreement with this we have not observed a purely homeostatic effect of *Smed-hh(RNAi)* or *Smed-ptc(RNAi)* without inducing regeneration by amputation and two previous studies did not report homeostatic effects on the A/P axis [16], [17].

Both two tailed *Smed-APC-1(RNAi)* and *Smed-ptc(RNAi)* animals homeostatically regenerate brain tissue around new pharynges (Figure 7 E-J). In addition in *Smed-ptc(RNAi)* animals anterior regeneration takes place without further wounding from central body regions and the new pharynx re-orientates (see Figure 7G and J). Interestingly, on re-examination of previously published data we see some evidence that this phenotype was generated but not clearly observed or described, for example Figure 3E in Gurley *et al*. [12] shows distinct thickening on the VNCs at the level of the ectopic pharynx in *Smed-APC-1(RNAi)* animals. The implications of these observations are twofold. Firstly, it suggests that in the absence of anterior fate the new and old pharynx represent the most anterior tissues and may instruct pASC to brain neuron differentiation. Evidence in support of this possibility includes the observation that *Smed-prep*, required for anterior regeneration, and *Smed-sFRP-1* a marker of anterior fate, without as yet an ascribed function, are also expressed around and in the pharyngeal region [29]. Secondly, it suggests that high global levels of Wnt signalling after *Smed-APC-1(RNAi)* (Figure 7F) prevent the brain structures formed around pharynges from elaborating to form an outgrowth and a new anterior (Figure 7I). However, in *Smed-ptc(RNAi)* animals a gradient of Wnt activity appears to be restored (Figure 7G), with high Wnt signals at both poles and low Wnt signals in central body regions. This in turn allows outgrowth of the brain and anterior structures and formation of a complete new A/P axis (Figure 7J). In agreement with this model we observed that double *Smed-APC-1(RNAi);Smed-ptc(RNAi)* animals phenocopy *Smed-APC-1(RNAi)* animals and form ectopic peri-pharyngeal brain structures but not anterior outgrowths and a new A/P axis (Figure 5). We would suggest that this difference between *Smed-ptc(RNAi)* and *Smed-APC-1(RNAi)* animals is mediated at the level of *Smed-prep* activity in the region around the pharynx, but this remains to be formally tested (Figure 5 J–L).

Future work will be focused on identifying the molecular components that control the early Wnt/Hh independent gradient of anterior regeneration rates and early phase of brain regeneration we describe here. Clues to what these might be exist in the data presented here and in the findings of previous studies. It has been previously shown that loss of *ndk* leads to expansion of brain tissues without affecting other A/P fates [30], and that this expansion is suppressed by concomitant knockdown of two FGF-receptors expressed in stem cells [30], [31]. This implicates FGF-receptors in receiving an unknown signal for which *ndk* is somehow responsible and has led to the Brain Activator hypothesis of brain regeneration [31]. Here, we have not studied the role of *ndk* or FGF-receptors in early brain regeneration directly. However, we do observe that in *Smed-prep(RNAi)* animals early brain structures are smaller than in *Smed-ptc(RNAi)*, *Smed-APC-1(RNAi)* or HU treated animals. Given that one role of Smed-Prep is to allow pASC progeny to form brain tissue in the suppressive presence of ndk [19], we interpret small early brain structures in *Smed-prep(RNAi)* animals as indirect evidence that ndk is active at this early stage of brain structure differentiation, and is suppressing brain fates. However, whether *ndk* and FGF signalling will also be implicated in controlling anterior regeneration rates remains to be tested. Another possible clue as to the Wnt independent mechanisms controlling anterior regenerative rate and early brain regeneration comes from work on gap junction function [32]. Oviedo *et al,* demonstrated that either intact VNC connections to the brain or active gap junction signalling are required to limit anterior regeneration to the anterior rather than all amputation sites. In this scenario it is tempting to speculate that the requirement for intact VNC to brain connections or functioning gap junctions reflects the need for an active form of the mechanism mediated by *ndk* to inhibit anterior fates. These ideas await direct testing with combinatorial RNAi and gap junction blockers [32].

In summary we reveal an early phase of brain regeneration that relies on stem cells through S-phase and suggests for the first time that pASCs may respond to regenerative signals differentially according to their position in the cell cycle. This early phase may be functionally redundant with respect to regenerative outcomes as it is not required for posterior brain structures induced by abrogation of Wnt signalling. We show that the roles of Hh and Wnt signalling are not equivalent during regeneration or homeostasis of posterior fates and that planarians can remodel their A/P axis to produce anterior tissues from central body regions given an intact Wnt signalling gradient. Overall we have revealed previously unappreciated but important layers of complexity in the A/P and brain regeneration processes.

## Materials and Methods

### Planarian culture

Asexual *Schmidtea mediterranea* were fed organic veal liver and starved for at least one week prior to experiments or amputation. The animals were not fed for the duration of the experiments.

### Experimental manipulations of planarians

All animals used in experiments to investigate the anterior regeneration rate were 1.5 cm in length (Figure 1). Animals were measured with a graticule down a binocular dissection microscope while relaxed on card on a layer of ice to allow accurate measurement. Animals were cut as shown on in the diagrams referred to in the text using fragments of shattered double edged razor blades (Wilkinson Sword, UK) clipped into a dental micro clamp (World Precision Instruments). Animals were cut while relaxed on ice in 15 minute batches and fragments allowed to regenerate at 20°C for observation of eye regeneration or before fixation at the required time point. For serial amputation along the A/P axis a graticule was used to aid in accurate positioning of the cuts to produce equally sized and equivalently positioned fragments for pieces 2–7 (Figure 1A). For generation for pre- and post-pharyngeal fragments cuts were place just anterior and posterior to the pharynx, with further amputations to the anterior (pre-pharyngeal) and posterior (post-pharyngeal) to generate fragments 1.4–1.5 mm in length. Any fragments that did not meet these limits were disregarded.

For RNAi experiments all animals were 1.3 cm (to the nearest 500 µm) in length to control for the any effect of size on overall regeneration rates. In these experiments pre- and post-pharyngeal fragments were kept within the same size limits (∼1.4 mm) as experiments investigating anterior regeneration rate in wild type animals.

### RNAi Experiments and phenotypic scoring

The *Smed-APC-1, Smed-ptc, Smed-βcatenin-1* and *Smed-prep* sequences have previously been submitted to GenBank with accession numbers EU130785, GQ337475, EU082826 and GU290186 respectively. Double stranded RNA (dsRNA) was produced as previously described for *Smed-APC-1*, *Smed-ptc, Smed-βcatenin-1, Smed-prep* and a control fragment of *dsRed*, which has no homology to the planarian genome [19], [33], [34]. RNAi was performed by injected 3×32 nl of 0.5–2 µg/µl dsRNA, depending on the experiment, for each day of injection as described previously [19], [34]. For injection plans see Figure S1A. Animals were amputated (see schematics, legends and above) to observe regeneration. Animals were scored for differences compared to *dsRed(RNAi)* controls over the course of up to several weeks. Animals were observed and bright field images were taken on a Zeiss Discovery V8 from Carl Zeiss using Axio Cam MRC from Carl Zeiss. All scoring of photoreceptor regeneration rates was performed blind and bright field images taken to allow later independent blind scoring by a second individual. No differences in scoring for individual images were detected.

### In situ hybridisation

Whole mount *in situ* hybridisation was carried out on regenerating pieces and were fixed and stained using methods previously described [35]. The following probes were used; *Smed-sFRP* [11], [14], *Smed-GluR*, *Smed-Gpas* [28] the homologue of clone 1791_HH from *Dugesia japonica*, which specifically labels the brain lateral branches and the pharyngeal cuff [36], *H.10.2f* [37] and *Smed-porcupine1* [12]. Bright field images were taken on a Zeiss Discovery V8 from Carl Zeiss using Axio Cam MRC from Carl Zeiss.

### Immunohistochemistry

For immune-staining animals were killed, fixed and processed as previously described [19], [35], [38]. The worms were stained with the following primary antibodies; anti-SYNORF1, a mouse monoclonal antibody specific for synapsin (Developmental Studies Hybridoma Bank, dilution 1:100), and anti-H3P-Ser10, a rabbit monoclonal antibody which labels mitotic cells (Upstate Millipore, dilution 1:1000). The following secondary antibodies were used: goat anti-mouse antibody conjugated to Alexa 488 (Molecular Probes, used at 1:1000 dilution), and goat anti-rabbit antibody conjugated to Alexafluor 568 (Molecular Probes, dilution 1:1000) respectively. Fluorescent images were taken on a Leica MZ16F fluorescence stereomicroscope using Leica DFC 300Fx camera (Leica Lasertechnik, Heidelberg).

### Analysis of proliferation

To assess proliferation, animals were stained with anti-phospho-Serine10 histone H3 as described above. For each regenerating piece or homeostatic worm the number of mitotic cells and the area in mm^2^ was determined using Adobe Photoshop CS4. The average number of proliferative cells per mm^2^ was calculated from pooled samples at each regeneration and homeostasis time point.

### Drug treatment

Hydroxyurea (Sigma) was used as previously described [39] at a 20 mM concentration dissolved in normal planarian water. Animals were treated with HU for 15 hours prior to cutting and cultured in HU for the remainder of the experiment. Colchicine (Sigma) was used at 5mM dissolved in normal planarian water. Animals were treated with Colchicine for 2 hours prior to cutting and cultured in colchicine for the remainder of the experiment.

### FACS analysis of stem cells and stem cell progeny

Planarian cell dissociation was performed following a protocol based on that previously used [40]. Briefly, the mucose was removed from the animals by treatment with 2% L-cysteine hydrochloride monohydrate (Merck), pH 7.2. They were subsequently cut into small pieces, transferred into CMF buffer containing papain (Worthington) and mechanically dissociated. The cells were then stained for 2 hours with 10 µg/ml Hoechst 33342 (Sigma). Propidium iodide (Sigma) was added at concentration of 1 µg/ml for FACS analysis (Coulter Ultra Flow).

## Supporting Information

Figure S1 **Schematic of RNAi experiments.** For all experiments animals were injected on each of three consecutive days, followed by a 4 days break, and then three further days of injection. On each day 3 injections of 32 nl were applied. Animals were amputated as described in the text 10 days after initial injection and observed
(PDF)

Figure S2 **Controls for Smed-sFRP-1 expression.** Control experiments showing that (A) *Smed-APC-1(RNAi)* and (B) *Smed-ptc(RNAi)* animals maintain *Smed-sFRP-1* expression in the anterior of head fragments even though expression is absent from anterior amputation sites (Figure 2).
(PDF)

Figure S3 **Proliferation in regenerating pre- and post- pharyngeal fragments.** In order to test if differences in the timing of anterior regeneration along the A/P axis were due to proliferation we counted mitotic cells in these fragments in control (dsRed injected) and experimental animals. Only Smed-ptc(RNAi) animals showed significant effects (previously reported in [16]). From this we conclude that differences in anterior regenerative rate are not a result of changes in proliferation.
(PDF)

Figure S4 **Ultimate Regeneration of two tails in** ***Smed-APC-1(RNAi)*** **and** ***Smed-ptc(RNAi)*** **animals.** In situ analysis with Smed-porcupine-1 in (A) control and experimental animals to show that both (B) *Smed-APC-1(RNAi)* and (C) *Smed-ptc(RNAi)* animals regenerate two tails with two characteristic major posterior branches as previously reported [12], [16], [17]. Controls animals regenerate a normal tail and an anterior with single major gut branch (A). Scale bar represent 250 µm.
(PDF)

Figure S5 **Expression of the posterior marker Smed-Fz-4 in** ***Smed-APC-1(RNAi)*** **and (B)** ***Smed-ptc(RNAi)*** **animals.** (A,B) The expression of the posterior marker Smed-Fz-4 is localized to the posterior of control injected animals. (C,D) Expression in *Smed-APC-1(RNAi)* and (E,F) *Smed-ptc(RNAi)* animals is expanded as posterior fate expands in these animals. Scale bar represent 250 µm.
(PDF)

Figure S6 **Formation of peri-pharyngeal brain structure in** ***Smed-APC-1(RNAi)*** **animals.** (A) All regenerating truck fragments form *Smed-GluR* (shown) or *Smed-Gpas* positive peri-pharyngeal brain structures (see Figure 5). (B) In addition 76% also form brain structures around the old pharynx.
(PDF)

Figure S7 **Formation of a new anterior and A/P axis in** ***Smed-ptc(RNAi)*** **worms.** (A–D) All *dsRed(RNAi)* control worms and *Smed-ptc(RNAi)* head fragments regenerate normally while (E,F) *Smed-ptc(RNAi)* trunk and tail fragments initially regenerate tails. (K,L) By 14dR *Smed-ptc(RNAi)* animals begun to develop distinct centrally positioned outgrowths, (Q,R) These outgrowths progress and (W,X) eventually form a new head with photoreceptors and a brain (Figure 5).
(PDF)

Figure S8 **Schematic explaining double cut experiments.** Trunk pieces were amputated as depicted and both early blastemas re-amputated after regeneration had been allowed to proceed for a set time. These animals were then stained with Smed-Gpas, and Smed-GluR to assay early brain formation events.
(PDF)

Figure S9 **Hydroxyurea treatment leads to depletion of cycling cells in G2 and M phase**. (A–K) Control animals maintain cells in G2 and M phase and pASCs continue to cycle over the first 72hR. (L–V) Treatment with HU before amputation leads to a significant depletion of cells progressing through S-phase (blue box in plot M) and results in eventual depletion of G2 and M cells, as these compartments fail to be renewed.
(PDF)

Figure S10 **Control in situ hybridization for HU treated** ***Smed-APC-1(RNAi)*** **and** ***Smed-ptc(RNAi)*** **worms.** Control in situ hybridization images of *Smed-GluR* expression in head fragments of e(A) *Smed-APC-1(RNAi)* and (B) *Smed-ptc(RNAi) worms.*
(PDF)

Figure S11 **Effect of colchicine treatment on brain regeneration.** Colchicine blocks cells transiting M phase and also blocks early brain regeneration (B) that proceeds normally in control worms (A). We cannot rule out that the effect of colchicine on brain regeneration is through blocking the differentiation of post-mitotic pASC progeny. Scale bars 100 µm.
(PDF)
