# Supplementary figures and images for: Combining Classical and Molecular Approaches Elaborates on the Complexity of Mechanisms Underpinning Anterior Regeneration
Source: PLoS One. 2011 Nov 18;6(11):e27927. doi: 10.1371/journal.pone.0027927 (PMC3220713; doi:10.1371/journal.pone.0027927)

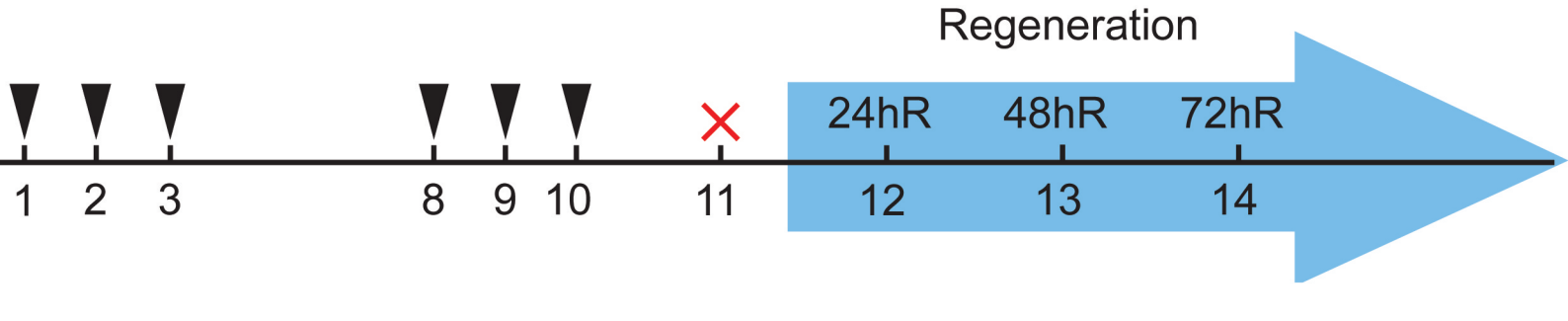

Supplement: Figure S1 — Schematic of RNAi experiments. For all experiments animals were injected on each of three consecutive days, followed by a 4 days break, and then three further days of injection. On each day 3 injections of 32 nl were applied. Animals were amputated as described in the text 10 days after initial injection and observed (PDF) [file pone.0027927.s001.pdf]

APC-1(RNAi)

*ptc*(RNAi)

**A**

**B**

48hR

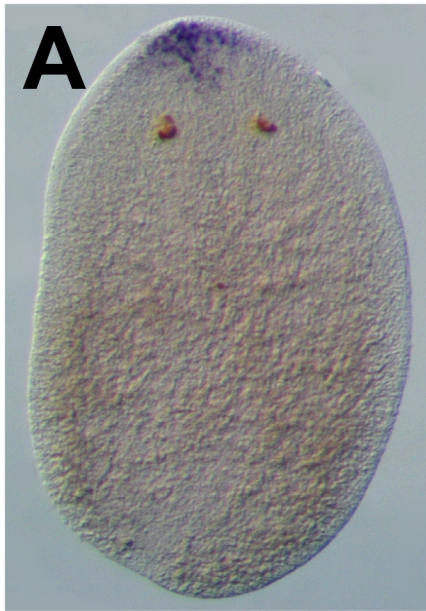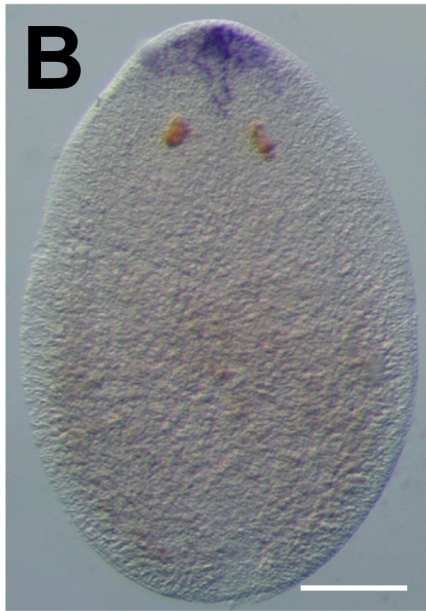

Supplement: Figure S2 — Controls for Smed-sFRP-1 expression. Control experiments showing that (A) Smed-APC-1(RNAi) and (B) Smed-ptc(RNAi) animals maintain Smed-sFRP-1 expression in the anterior of head fragments even though expression is absent from anterior amputation sites (Figure 2). (PDF) [file pone.0027927.s002.pdf]

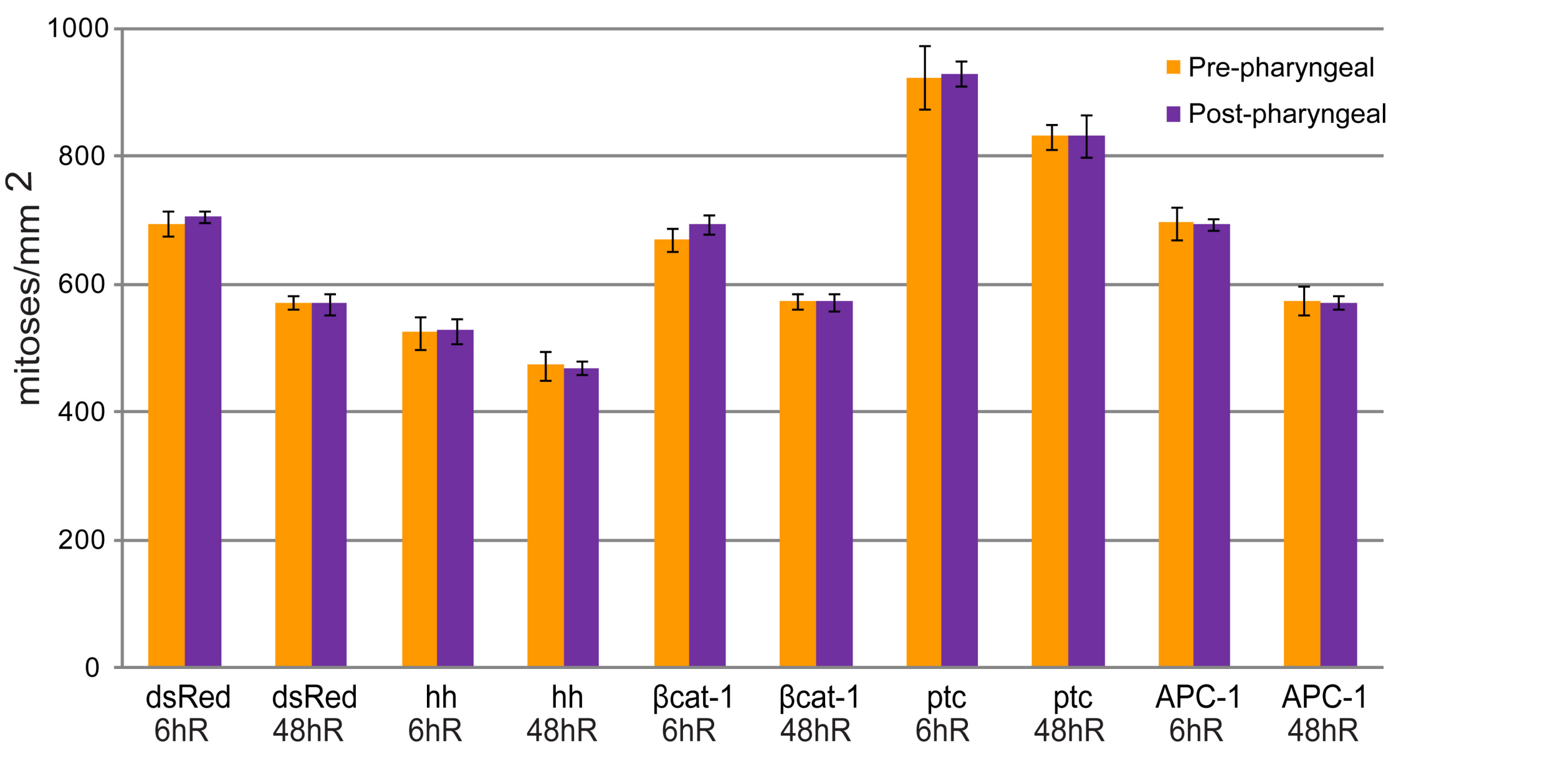

Supplement: Figure S3 — Proliferation in regenerating pre- and post- pharyngeal fragments. In order to test if differences in the timing of anterior regeneration along the A/P axis were due to proliferation we counted mitotic cells in these fragments in control (dsRed injected) and experimental animals. Only Smed-ptc(RNAi) animals showed significant effects (previously reported in [16]). From this we conclude that differences in anterior regenerative rate are not a result of changes in proliferation. (PDF) [file pone.0027927.s003.pdf]

ptc(RNAi)      APC-1(RNAi)      dsRed(RNAi)

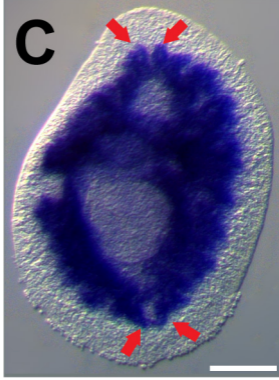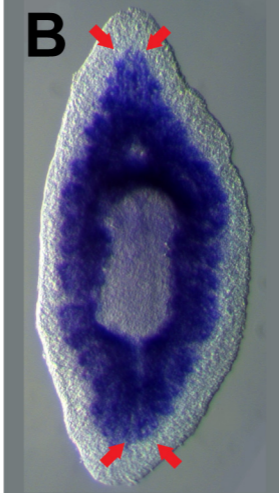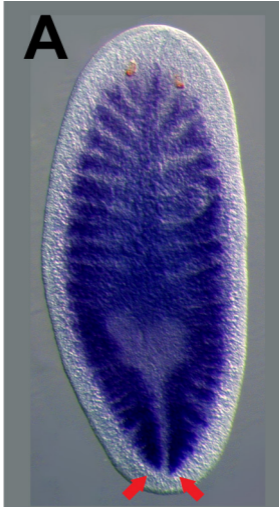

porcn-1

Supplement: Figure S4 — Ultimate Regeneration of two tails in Smed-APC-1(RNAi) and Smed-ptc(RNAi) animals. In situ analysis with Smed-porcupine-1 in (A) control and experimental animals to show that both (B) Smed-APC-1(RNAi) and (C) Smed-ptc(RNAi) animals regenerate two tails with two characteristic major posterior branches as previously reported [12], [16], [17]. Controls animals regenerate a normal tail and an anterior with single major gut branch (A). Scale bar represent 250 µm. (PDF) [file pone.0027927.s004.pdf]

dsRed(RNAi)

APC-1(RNAi)

*ptc*(RNAi)

48hR

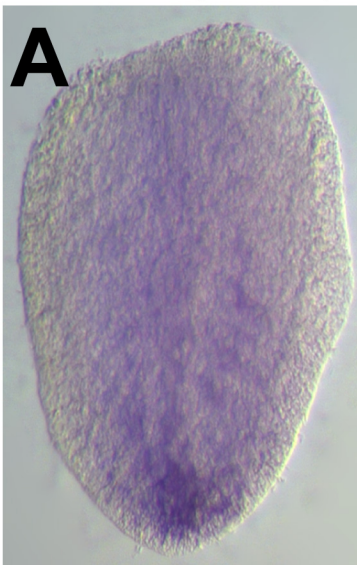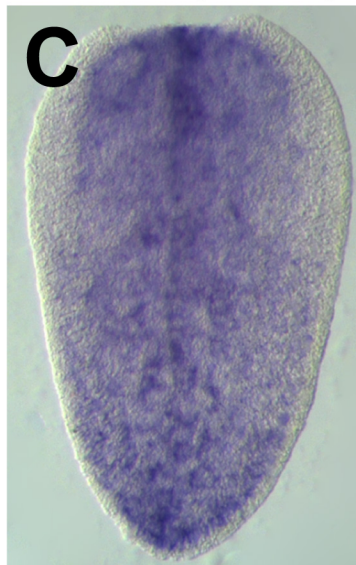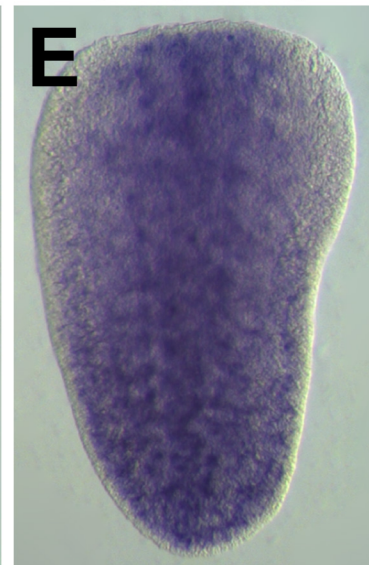

72hR

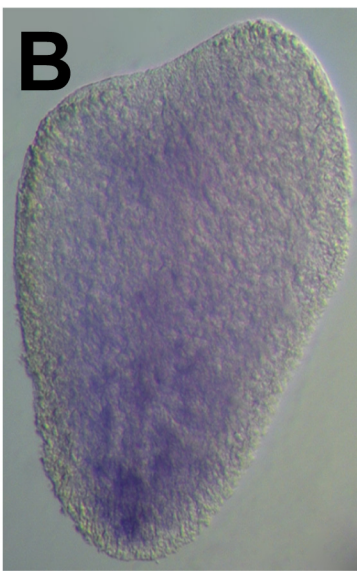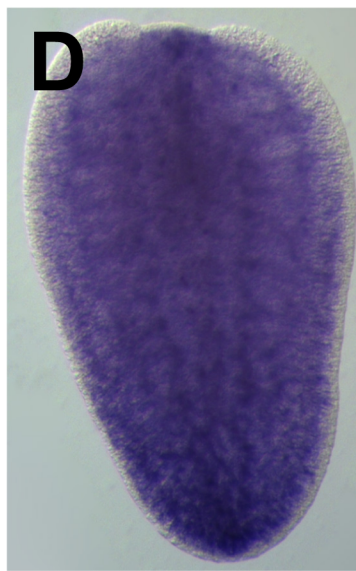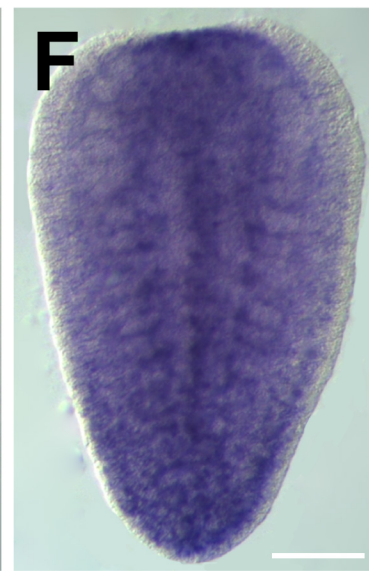

Supplement: Figure S5 — Expression of the posterior marker Smed-Fz-4 in Smed-APC-1(RNAi) and (B) Smed-ptc(RNAi) animals. (A,B) The expression of the posterior marker Smed-Fz-4 is localized to the posterior of control injected animals. (C,D) Expression in Smed-APC-1(RNAi) and (E,F) Smed-ptc(RNAi) animals is expanded as posterior fate expands in these animals. Scale bar represent 250 µm. (PDF) [file pone.0027927.s005.pdf]

# APC-1(RNAi)

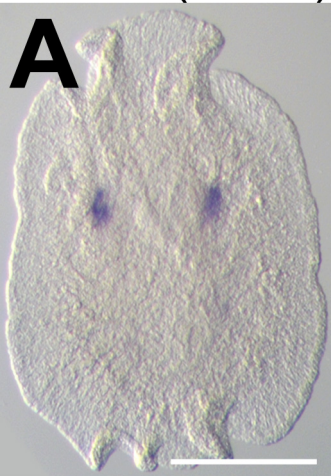

24%

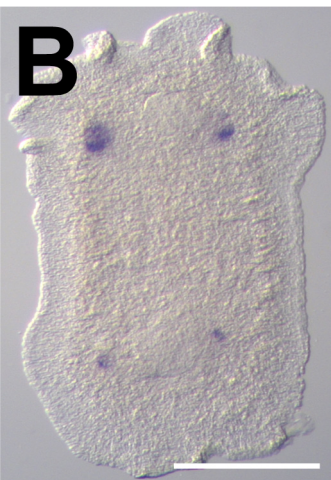

76%

Supplement: Figure S6 — Formation of peri-pharyngeal brain structure in Smed-APC-1(RNAi) animals. (A) All regenerating truck fragments form Smed-GluR (shown) or Smed-Gpas positive peri-pharyngeal brain structures (see Figure 5). (B) In addition 76% also form brain structures around the old pharynx. (PDF) [file pone.0027927.s006.pdf]

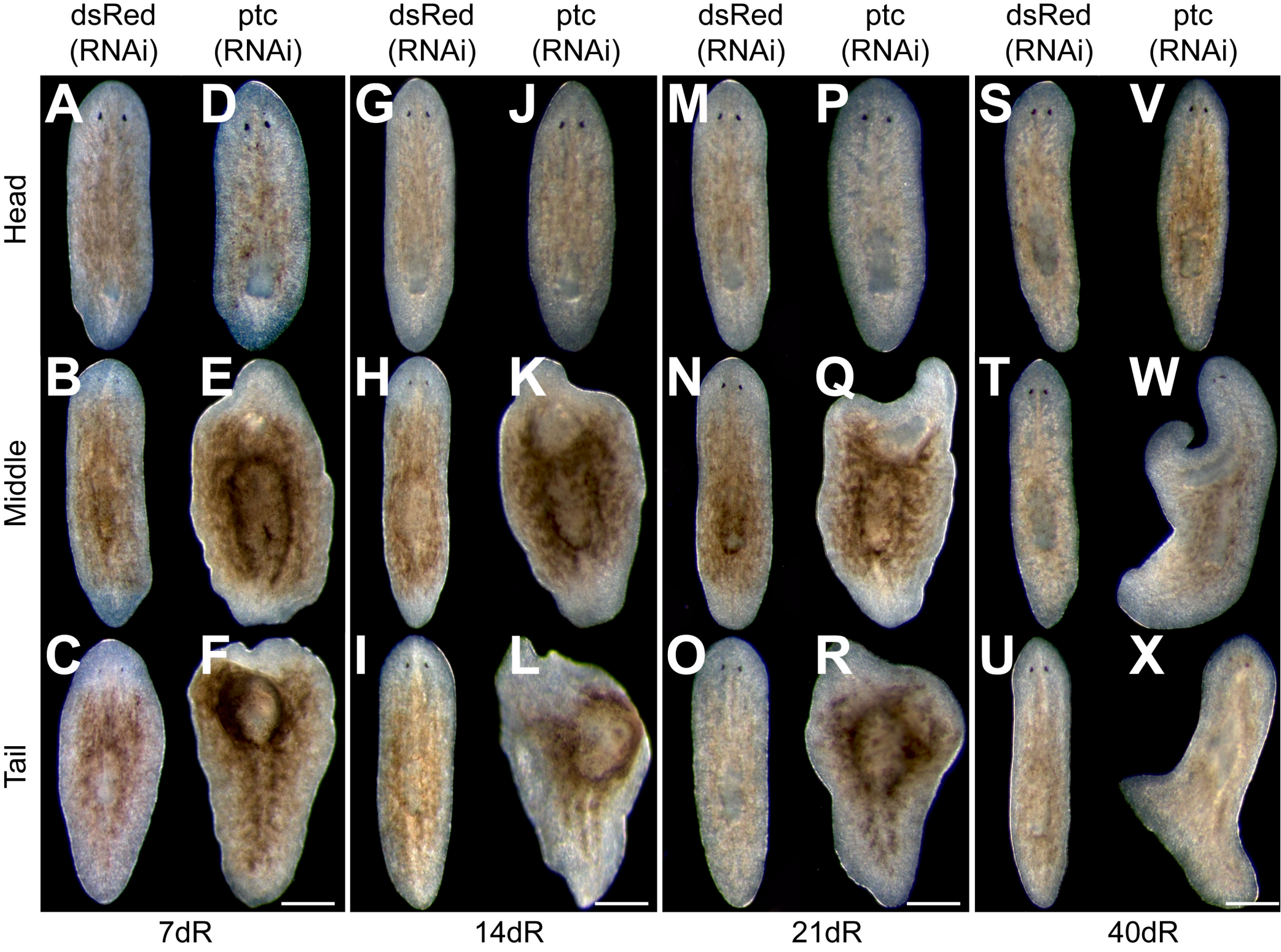

Supplement: Figure S7 — Formation of a new anterior and A/P axis in Smed-ptc(RNAi) worms. (A–D) All dsRed(RNAi) control worms and Smed-ptc(RNAi) head fragments regenerate normally while (E,F) Smed-ptc(RNAi) trunk and tail fragments initially regenerate tails. (K,L) By 14dR Smed-ptc(RNAi) animals begun to develop distinct centrally positioned outgrowths, (Q,R) These outgrowths progress and (W,X) eventually form a new head with photoreceptors and a brain (Figure 5). (PDF) [file pone.0027927.s007.pdf]

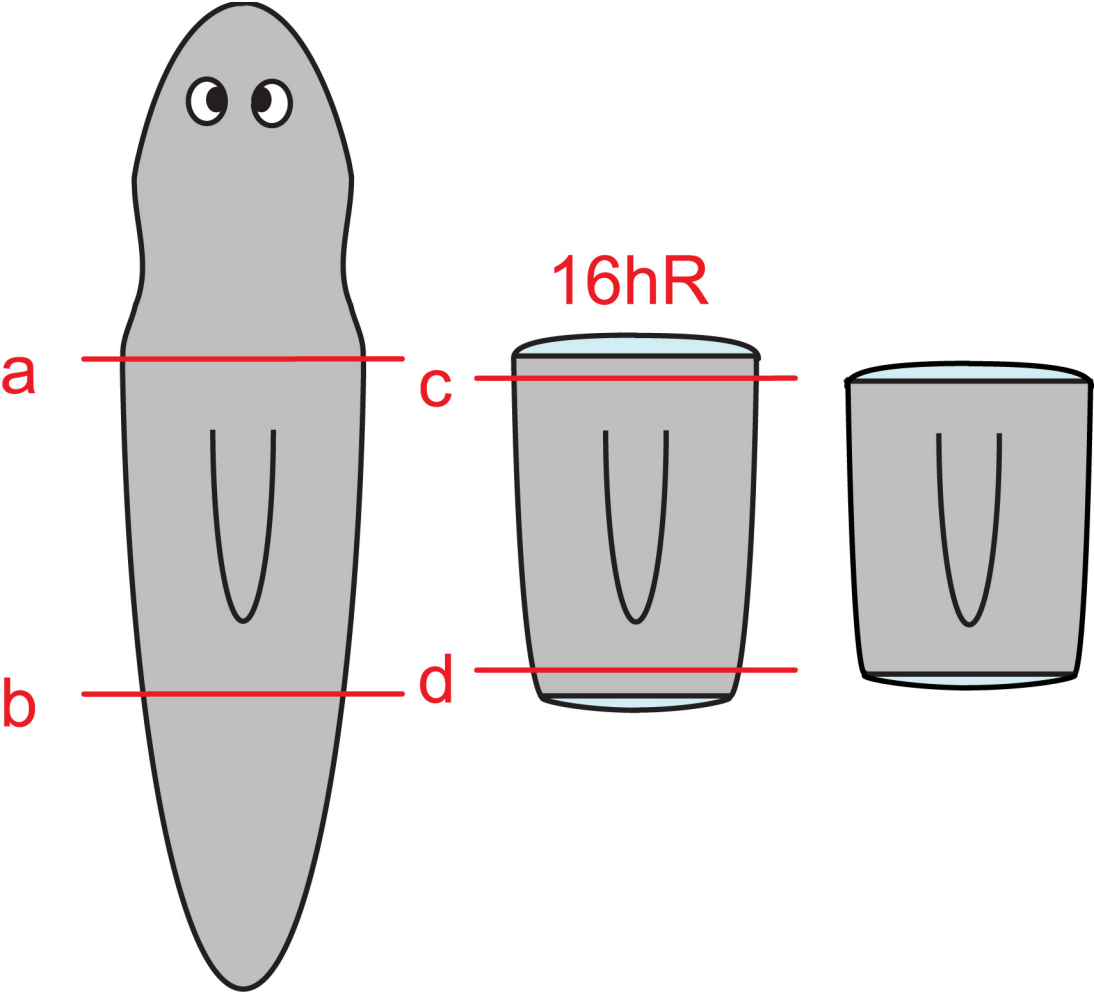

Supplement: Figure S8 — Schematic explaining double cut experiments. Trunk pieces were amputated as depicted and both early blastemas re-amputated after regeneration had been allowed to proceed for a set time. These animals were then stained with Smed-Gpas, and Smed-GluR to assay early brain formation events. (PDF) [file pone.0027927.s008.pdf]

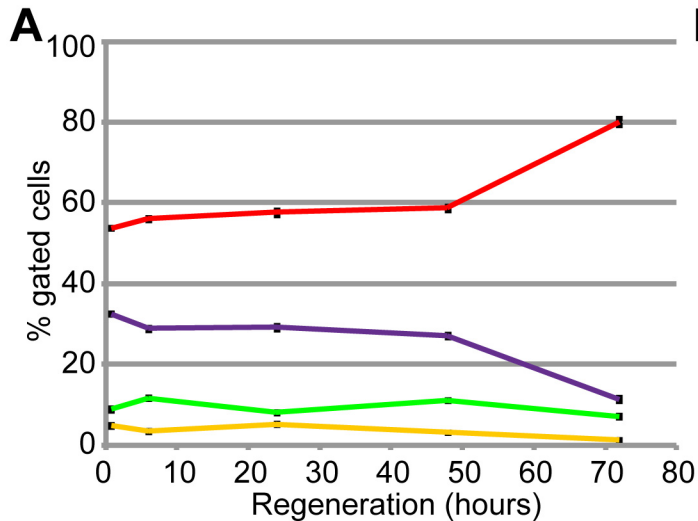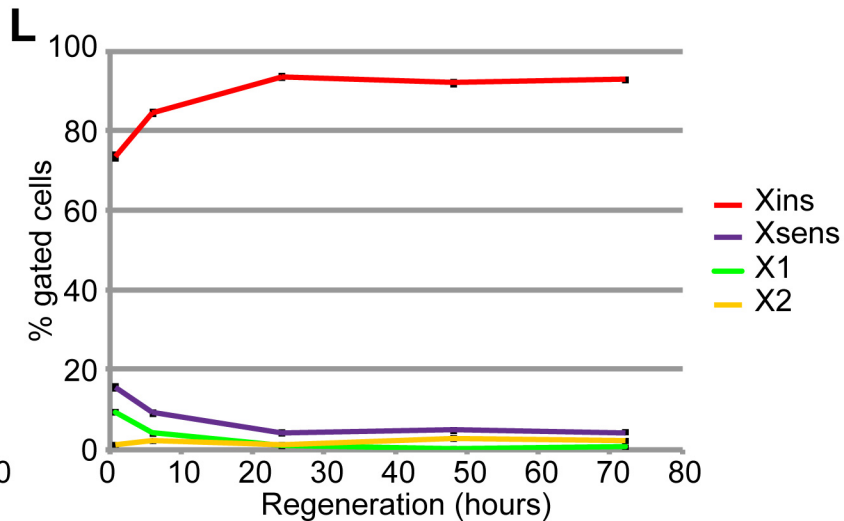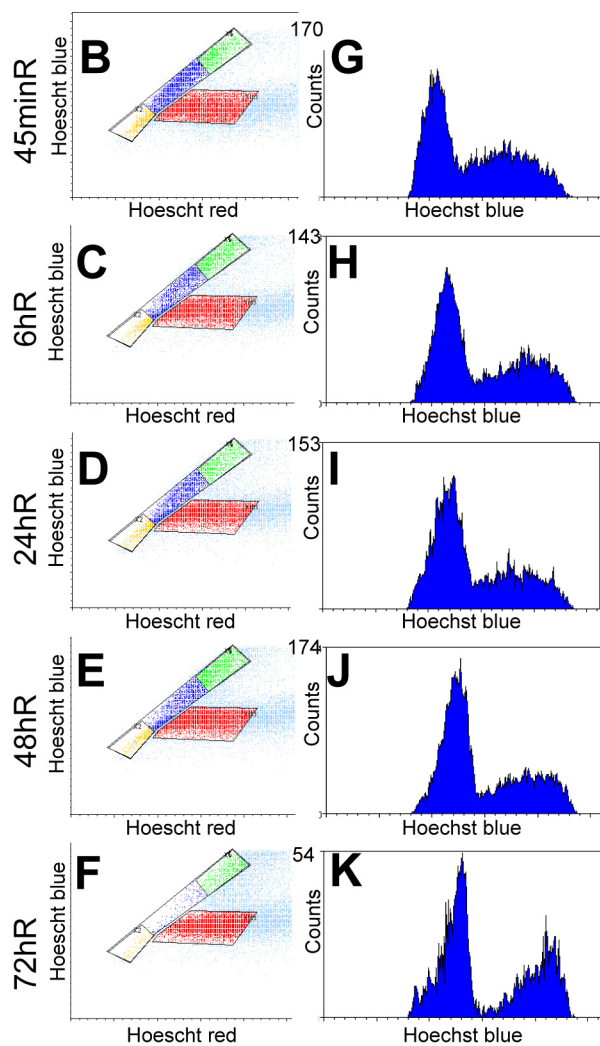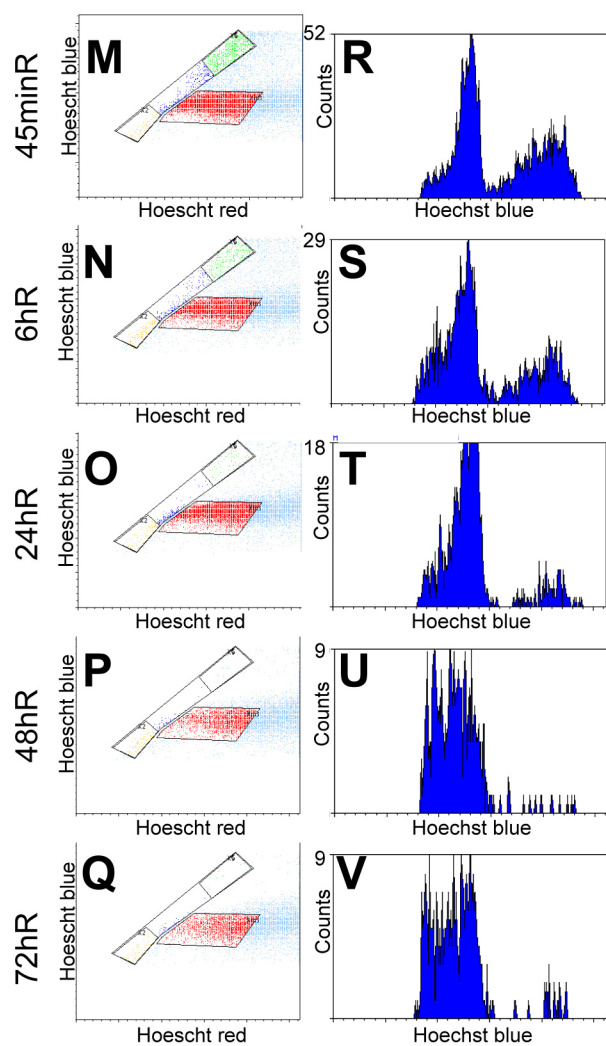

Supplement: Figure S9 — Hydroxyurea treatment leads to depletion of cycling cells in G2 and M phase. (A–K) Control animals maintain cells in G2 and M phase and pASCs continue to cycle over the first 72hR. (L–V) Treatment with HU before amputation leads to a significant depletion of cells progressing through S-phase (blue box in plot M) and results in eventual depletion of G2 and M cells, as these compartments fail to be renewed. (PDF) [file pone.0027927.s009.pdf]

APC-1(RNAi)

*ptc*(RNAi)

**A**

**B**

GluR

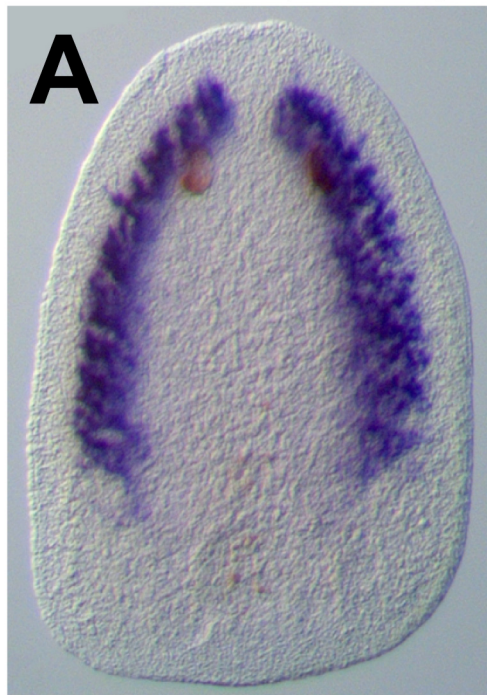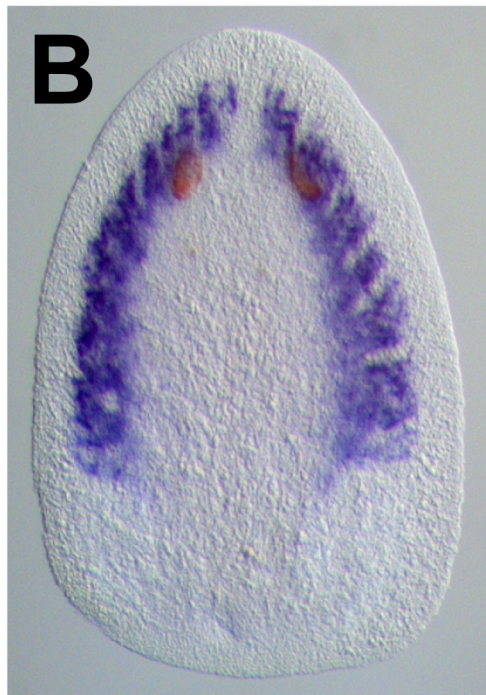

72hR

72hR

Supplement: Figure S10 — Control in situ hybridization for HU treated Smed-APC-1(RNAi) and Smed-ptc(RNAi) worms. Control in situ hybridization images of Smed-GluR expression in head fragments of e(A) Smed-APC-1(RNAi) and (B) Smed-ptc(RNAi) worms. (PDF) [file pone.0027927.s010.pdf]

# Colchicine

**A**

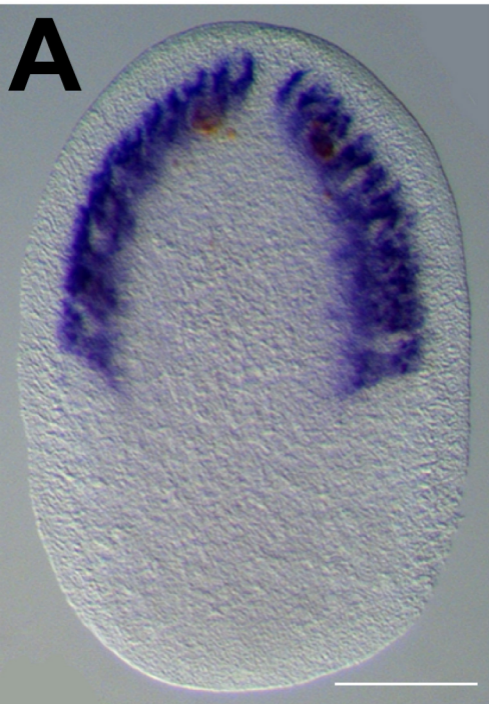

**B**

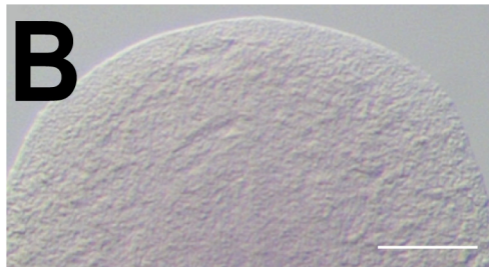

72hR

Supplement: Figure S11 — Effect of colchicine treatment on brain regeneration. Colchicine blocks cells transiting M phase and also blocks early brain regeneration (B) that proceeds normally in control worms (A). We cannot rule out that the effect of colchicine on brain regeneration is through blocking the differentiation of post-mitotic pASC progeny. Scale bars 100 µm. (PDF) [file pone.0027927.s011.pdf]
